# What is the Impact of Endothelial-to-Mesenchymal Transition in Solid Tumours: A Qualitative Systematic Review and Quantitative Meta-Analysis

**DOI:** 10.7150/ijbs.107045

**Published:** 2025-02-24

**Authors:** Pablo Hernández-Camarero, Belén Toledo, Ana Belén Diaz-Ruano, Aitor González-Titos, María Belén García-Ortega, Macarena Perán

**Affiliations:** 1 Department of Health Sciences, University of Jaén, Campus de las Lagunillas, E-23071 Jaén, Spain.; 2 Biopathology and Regenerative Medicine Institute (IBIMER), Centre for Biomedical Research, University of Granada, E-18016 Granada, Spain.; 3 Instituto de Investigación Sanitaria ibs. GRANADA, E-18071 Granada, Spain.; 4 Department of Cardiology, Virgen de las Nieves University Hospital, E-18071, Granada, Spain.; 5 Excellence Research Unit “Modeling Nature” (MNat), University of Granada, E-18016 Granada, Spain.

**Keywords:** Endothelial-to-mesenchymal transition, Tumour microenvironment, Metastasis, Endothelial cell, Cancer-associated fibroblasts, Meta-analysis, EndMT-related markers, Biomarkers, Therapeutic target

## Abstract

Endothelial-to-mesenchymal transition (EndMT) has gained increasing recognition as a crucial mechanism in the progression of solid cancers, influencing tumour heterogeneity, metastasis, and resistance to therapy. However, despite its growing importance, EndMT remains insufficiently studied within the cancer research landscape.

In this study, we conduct a systematic review, adhered to the 2020 PRISMA guidelines, of the existing literature on EndMT in solid tumours, examining its functional roles, key biomarkers, underlying mechanisms, experimental models, and potential as a target for therapeutic intervention. Our objective was to identify critical areas where further research is needed. In addition, we performed a meta-analysis to evaluate the variability in the expression of EndMT-related markers and their potential links to patient prognosis.

To this aim, literature searches were conducted in major databases including PubMed, Scopus, and Web of Science, covering studies published up to June 2024. The risk of bias of selected articles was evaluated using the OHAT tool, for the *in vitro* experiments and the SYRCLE tool for studies using animal models.

Out of an initial pool of 1,197 articles, 54 studies were selected for data extraction by two independent reviewers. Selected studies were identified according to specific inclusion/exclusion criteria applied through distinct stages like “title and abstract screening”, “full text article review” and “article bibliography screening”. Our analysis confirms that EndMT is a key contributor to tumour progression and metastasis, but several aspects remain poorly understood, particularly regarding the induction of EndMT in specific cancer types, its role in lymphatic endothelial cells, and its interactions with other stromal elements. We observed substantial heterogeneity in the biomarkers associated with EndMT, as well as variations in the endothelial cell types studied, the functional outcomes, and the molecular mechanisms involved. Our meta-analysis revealed significant variability in the expression of EndMT biomarkers, with notable correlations between changes in the expression of specific genes and patient outcomes, particularly in lung cancer.

In conclusion, it is essential for future research to focus on identifying the specific cancer and stromal cell types implicated in EndMT and to standardize endothelial cell models and protocols used for inducing EndMT. Investigating EndMT alongside well-established processes, such as epithelial-to-mesenchymal transition (EMT), and exploring its relationship with cancer-associated fibroblasts (CAFs) may provide valuable insights into its role in tumour biology and its impact on therapy resistance.

## Background

Research in cancer has made considerable progress in the past few decades, nevertheless each year its incidence and related mortality increases in certain tumour types, such as pancreatic cancer 1, melanoma 2 or uterine cancers 3, underscoring the ongoing limitations in our knowledge. Recently, the complexity of tumours, along with both inter- and intra-tumoral heterogeneity, has garnered increasing attention. It is now widely acknowledged that tumour growth and progression are not only driven by the cancerous cells themselves but also by the stromal components of the tumour microenvironment (TME), including cancer-associated fibroblasts (CAFs) 4, tumour-associated macrophages (TAMs) 5, and endothelial cells 6.

Among the many processes involved in cancer, the epithelial-to-mesenchymal transition (EMT) of tumour cells is one of the most extensively studied. EMT encompasses a dramatic transcriptional and phenotypic transformation in epithelial-like cancer cells, enabling them to acquire a migratory and pro-metastatic mesenchymal phenotype, exemplifying the remarkable plasticity of tumour biology 7. This molecular mechanism is closely linked to tumour progression, aggressiveness, drug resistance, and metastasis across a variety of cancers, including those of the liver 8, colon 9, lung 10, breast 11, and pancreas 12. Interestingly, stromal cells also undergo phenotypic alterations to support tumour growth, contributing to the TME in various ways. For example, CAFs often exhibit the overexpression of FAPα (fibroblast-associated protein alpha), a key marker linked to tumour progression and metastasis 13. Similarly, TAMs frequently undergo M2 polarization, a process that enhances their ability to promote immune suppression, tissue remodelling, and angiogenesis 14. These stromal modifications not only facilitate tumour cell proliferation but also enable the tumour to evade immune detection and resistance to therapeutic interventions, further complicating treatment strategies.

**Figure 1:**
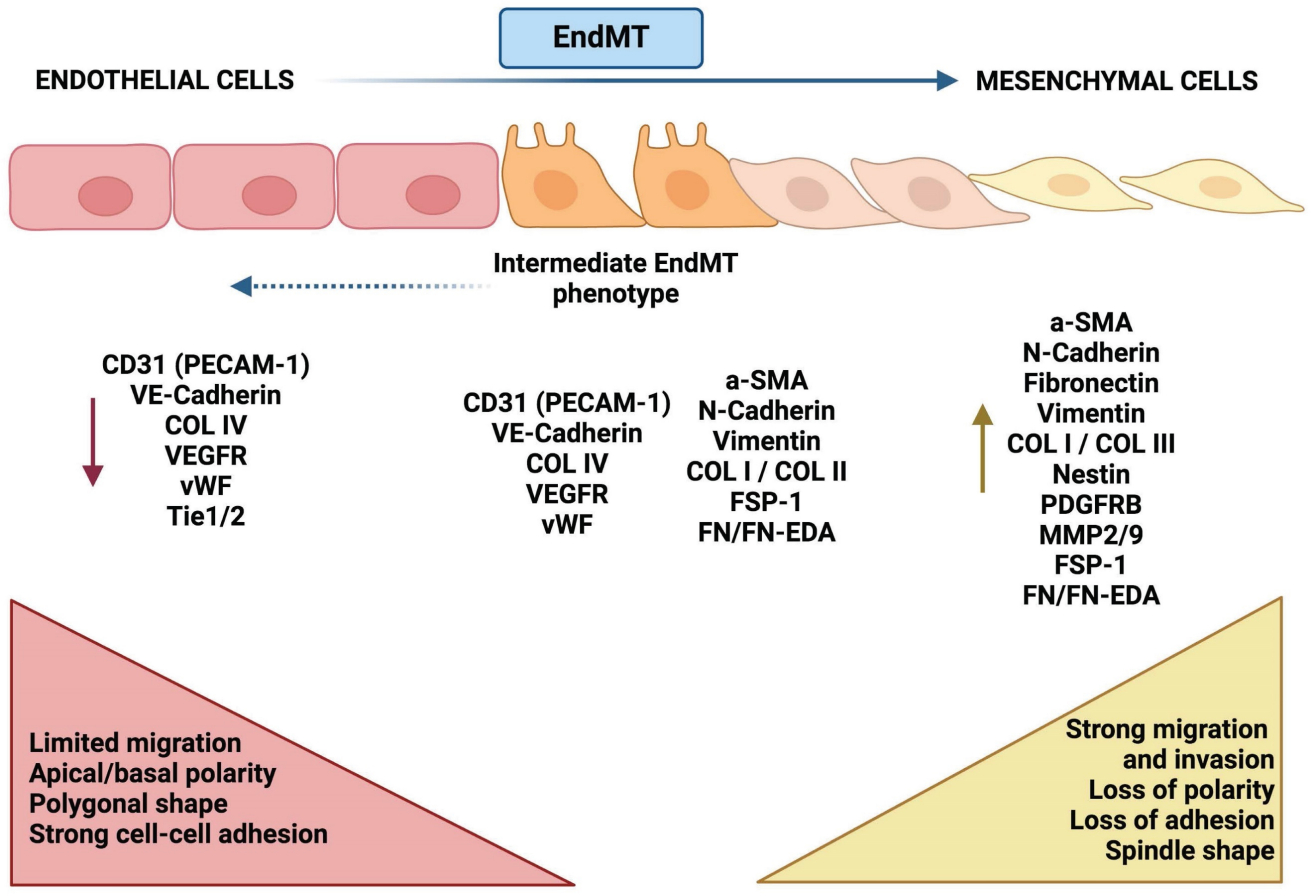
** Morphological and molecular changes during EndMT.** Endothelial-like cells (left, red) are typically characterized by tight intercellular connections, exhibiting a cobblestone morphology and expressing junctional proteins. Biomarkers such as VE-cadherin, CD31, and ZO-1 are indicative of a fully differentiated endothelial phenotype. In the transition phase (center, orange), these endothelial cells begin to lose their junctional integrity, undergoing morphological changes to assume a more spindle-like shape. This phase is characterized by the downregulation of endothelial markers (e.g., VE-cadherin, CD31) and the upregulation of mesenchymal-like markers (e.g., N-cadherin, vimentin), signaling the progression of the transition. In the mesenchymal phase (right, yellow), cells adopt a fibroblast-like phenotype, marked by increased motility and altered cell-cell adhesion. The predominant expression of mesenchymal markers (e.g., N-cadherin, vimentin, fibronectin), coupled with the loss of endothelial markers, indicates a complete switch in phenotype.

Endothelial-to-mesenchymal transition (EndMT) is a process similar to EMT, in which endothelial cells lose their characteristic endothelial features and acquire a mesenchymal-like phenotype. This transition has been observed in various pathological conditions, including fibrosis 15, cerebral cavernous malformations 16, atherosclerosis 17, vascular hypertension 18, and cancer 19. However, EndMT in the context of cancer is less well characterized than EMT. In fact, much of the research connecting endothelial cells to cancer has focused primarily on tumour angiogenesis, because this process is essential for providing the oxygen and nutrients required for tumour growth and metastasis 20. Consequently, research has largely concentrated on profiling this abnormal vasculature and developing therapeutic strategies aimed at normalizing tumour blood vessels 21. Nonetheless, growing experimental evidence underscores the broader role of endothelial cells in the TME beyond angiogenesis. For instance, angiocrine factors secreted by endothelial cells *(i.e. TIMP1 or IGFBP2)* have been shown to promote the proliferation, migration, and drug resistance of glioblastoma cells 20. Additionally, a strong association between endothelial cells and CAFs has been established, with CAFs in lung cancer playing a key role in the formation of aberrant vasculature in solid tumours by generating a pro-inflammatory environment with elevated concentrations of several cytokines, including TNFα, TGFβ1, IFNγ, IL1β, and MCP1 22. This connection is further supported by evidence that the phenotypic plasticity of endothelial cells, induced by EndMT, is critical for the generation of CAFs 23. In this same study, the EndMT process has also been linked to the progression of pancreatic cancer and melanoma, as well as to the increased fibrosis of the surrounding stroma. Actually, the EndMT process has been linked to the progression of pancreatic cancer and melanoma, as well as to the increased fibrosis of the surrounding stroma 23. Moreover, the endothelial plasticity driven by EndMT, marked by the upregulation of mesenchymal markers such as αSMA (α-smooth muscle actin), FSP1 (fibroblast-specific protein 1), N-Cadherin, and SNAI2, has been associated with the emergence of chemoresistance in malignant cells, including resistance to temozolomide and anti-VEGF antibodies in glioblastoma 24. Undeniably, the critical role of EndMT (increasing vascular permeability and enhancing trans-endothelial migration of metastatic cells) in various cancer-related processes, including tumour progression and metastasis, has been clearly demonstrated, particularly in melanoma and breast cancer 25. Figure 1 illustrates the phenotypic plasticity linked to EndMT, which enables endothelial cells to adapt to distinct signaling cues within the TME, positioning EndMT as a dynamic and pivotal player in tumour biology.

Given these insights, it is crucial to recognize the complexity and heterogeneity of EndMT across different tumour types. Understanding the molecular mechanisms driving EndMT and its interactions with the surrounding TME may be key to exploiting its plasticity as a therapeutic target. Therefore, the objective of this study was to conduct a qualitative systematic review to assess the current state of knowledge regarding EndMT in solid tumours. Specifically, we focused on experimental data examining the induction of EndMT in endothelial cells within the TME during tumour progression.

In this study, we explored into several key aspects of the EndMT, focusing on critical biomarkers associated with this process. We also identified several key areas that remain underexplored, suggesting promising avenues for further research to deepen our understanding of this complex phenomenon. These insights could potentially open the door to innovative therapeutic strategies aimed at targeting EndMT-related pathways in cancer. To this end, we conducted a comprehensive quantitative meta-analysis, examining the expression patterns of EndMT-associated markers across a range of solid tumours. This analysis also aimed to explore the relationship between these markers and tumour progression, providing valuable information on how EndMT contributes to cancer development and metastasis.

## Materials and Methods

### Search methodology and data analysis

This review is not officially registered. It was conducted in accordance with the 2020 Preferred Reporting Items for Systematic Reviews and Meta-Analyses (PRISMA) guidelines 26. Eligible publications focused on experimental data regarding the EndMT process in the context of solid tumours. Literature searches were performed using public databases such as PubMed, Scopus and Web of Science from inception until June 2024, employing the following search terms/strings: (“tumor*” OR “tumour*” OR “cancer*”) AND (“endothelial cell*” OR “EC” OR “tumor endothelial cell*” OR “tumour endothelial cell*” OR “cancer endothelial cell*” OR “tumor-derived endothelial cell*” OR “tumour-related endothelial cell*” OR “tumor-associated endothelial cell*” OR “tumour-derived endothelial cell*” OR “tumor-related endothelial cell*” OR “tumour-associated endothelial cell*” OR “cancer-derived endothelial cell*” OR “cancer-related endothelial cell*” OR “cancer-associated endothelial cell*” OR “TEC”) AND (“endothelial-mesenchymal transition*” OR “endothelial-to-mesenchymal transition*” OR “endothelial mesenchymal transition*” OR “endothelial to mesenchymal transition*” OR “EndMT*”).

**Figure 2:**
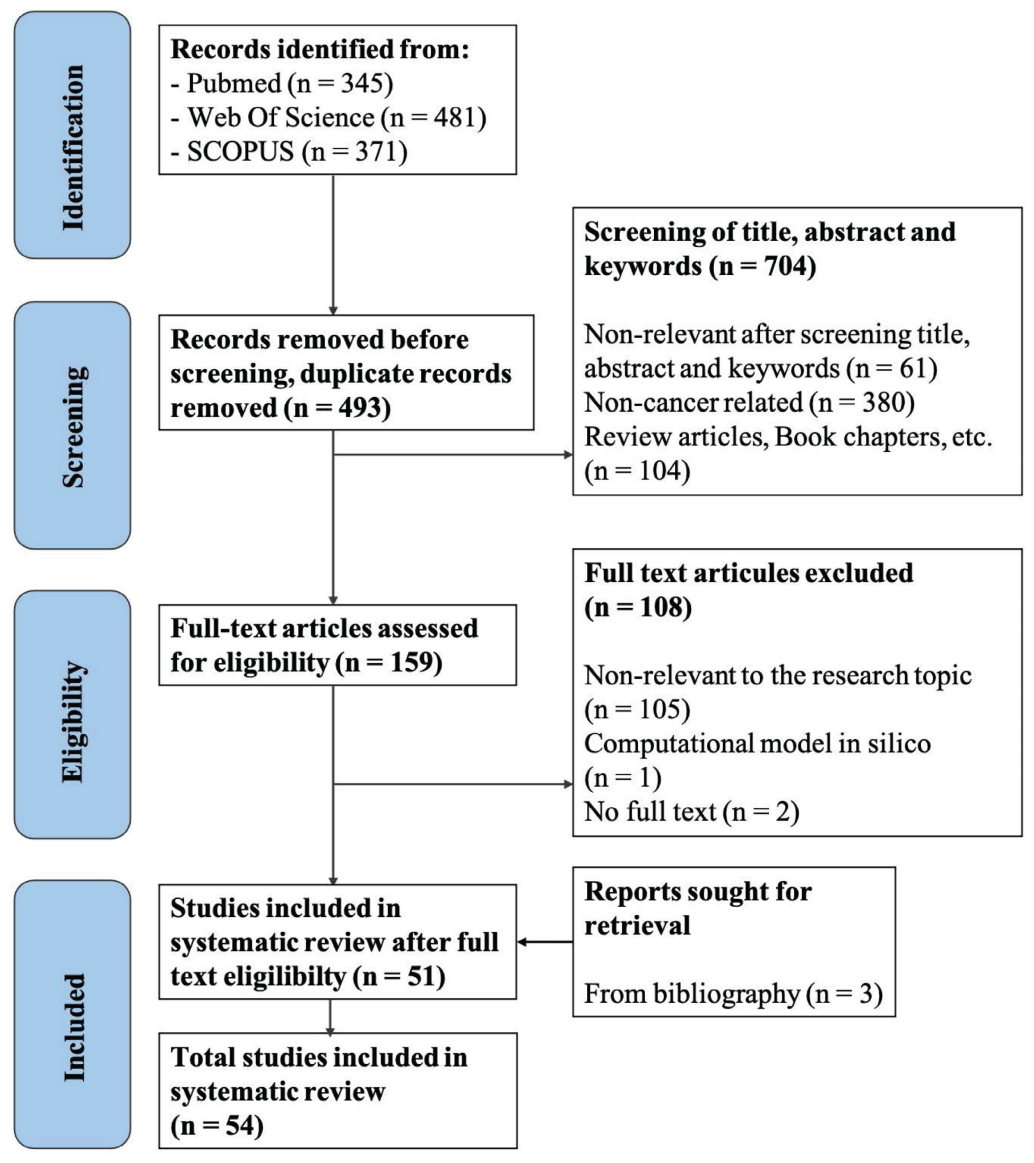
** Flowchart of study screening and selection process.** The screening process consists of four steps: identification, screening, eligibility assessment, and final inclusion.

The bibliographies of selected studies were also reviewed to identify additional relevant articles. Publications were screened for eligibility according to specific inclusion and exclusion criteria (Table 1). The screening protocol and outcomes are illustrated in Figure 2. In total, 1,197 articles were initially obtained, and 54 papers were ultimately selected for data analysis. The screening process was completed by two independent investigators, and any discrepancies were resolved according to the criteria set by the senior researcher. Data were extracted from the selected studies based on pre-agreed fields by two independent authors, with discrepancies resolved following the senior researcher's criteria.

### Risk of bias

Quality assessment of the selected *in vitro* studies was conducted using the Office of Health Assessment and Translation (OHAT) risk of bias rating tool. The assessment focused on the following criteria: identical experimental conditions, blinding of researchers, completeness of outcome data, exposure characterization, outcome assessment, complete reporting of outcomes, and other potential threats (e.g., statistical issues) 27. For studies using animal models, the risk of bias was evaluated using the Systematic Review Center for Laboratory Animal Experimentation (SYRCLE) tool 28. The assessment focused on the following criteria: sequence generation (selection bias), baseline characteristics (selection bias), allocation concealment (selection bias), random housing (performance bias), blinding (performance bias), random outcome assessment (detection bias), blinding (detection bias), incomplete outcome data (attrition bias), selective outcome reporting (reporting bias). Quality assessments were independently performed by two researchers, with any discrepancies resolved by the senior investigator. All eligible studies were included in the results synthesis regardless of their risk of bias rating. The visualization of the risk of bias assessment was performed using the Robvis tool 29.

### Meta-analysis

#### Analysis of Gene Expression Levels

For this meta-analysis, TCGA datasets corresponding to different cancer types were selected from UALCAN (http://ualcan.path.uab.edu), and specific biomarkers were evaluated for each cancer type. This analysis included both tumour samples and normal tissue controls across various cancer types. The selection criteria focused on cancers with well-defined cohorts. The number of patients in each group (tumour vs. control) for each cancer type is detailed in Table 2.

For each cancer type, relevant biomarkers were selected based on their reported associations with cancer progression or prognosis. The primary metric used to summarize the data was the median expression level of the chosen biomarkers for each cancer type. Utilizing the median, rather than the mean, is particularly important in patient samples due to the presence of outliers and potential skewness in the data.

HeatMap representation was created by calculating the Log2(Fold Change) between tumour samples and controls. Statistical analyses were conducted using tools provided by the UALCAN platform, which facilitates comparisons between normal and tumour samples through non-parametric tests. The differences in gene expression levels between control and tumour groups were evaluated using the Mann-Whitney U test, a non-parametric method appropriate for comparing medians in data that do not follow a normal distribution. Statistical significance was set at thresholds of *p* < 0.05 (),* p* < 0.01 (), and *p* < 0.001 ().

### Survival analysis

To evaluate the association between gene expression levels and patient survival, Kaplan-Meier survival curves were generated using the KM Plotter web-based tool (https://kmplot.com/analysis/). This platform integrates gene expression data with survival information from various publicly available cancer datasets. For this study, we utilized the available datasets for colon cancer, breast cancer, lung cancer, and gastric cancer, without applying any exclusion criteria to the patient data.

Survival curves were plotted based on the median expression levels of selected genes of interest, dividing patients into two groups: high expression and low expression. Hazard ratios (HR), 95% confidence intervals (CI), and log-rank p-values were automatically calculated by the KM Plotter software and reported to assess statistical significance. All analyses were conducted using the default settings provided by the platform. The selected genes for this study are listed in Table 3.

## Results

### Literature screening protocol

The search strategy outlined in the methods section yielded a total of 1,197 articles. After removing duplicates, 704 articles remained for further screening. We then screened the titles and abstracts of these studies, selecting those that met the inclusion criteria (Table 1). This process resulted in 159 papers being chosen for full-text screening based on the specified inclusion/exclusion criteria. Following this final screening stage, 51 articles were selected for complete data extraction and analysis. Additionally, we reviewed the bibliographies of these selected studies to identify more eligible articles, resulting in the inclusion of three additional studies during the “retrieval” phase. Consequently, a total of 54 studies were included for data analysis in the systematic review. Figure 2 illustrates the details of the screening process and appendix A shows the list of the selected articles.

## 2. Risk of bias

**Figure 3:**
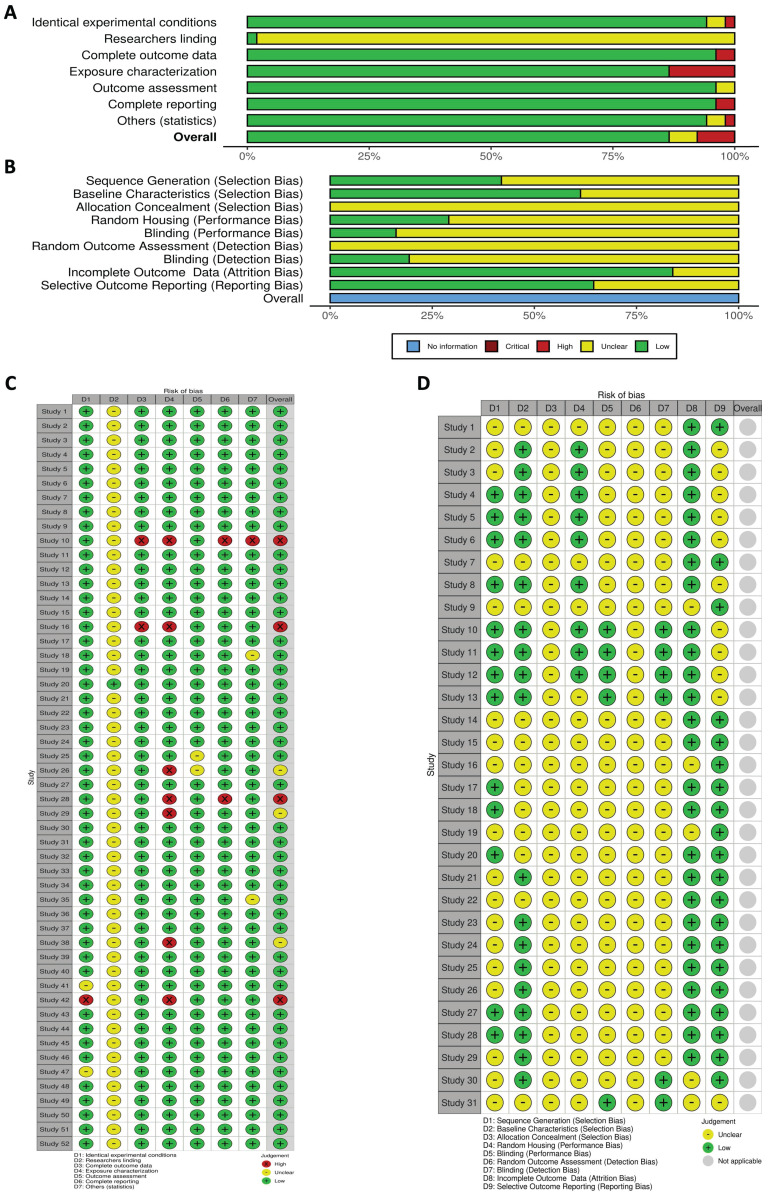
***In vitro* and* in vivo* risk of bias.** A: Results from the OHAT risk of bias rating tool assessing the risk of bias in the selected studies that included *in vitro* experiments. B: Risk of bias of selected articles that included *in vivo* studies based on animal models using the SYRCLE risk of bias tool. C and D display the independent assignment of risk of bias for each study, *in vitro* and *in vivo,* respectively. The graphics illustrate the distribution of bias risks across various study components, with categories ranging from low to high risk.

We evaluated the features of each study design by conducting a risk of bias assessment. The domain of bias referring "researcher blinding" in the *in vitro* studies could not be evaluated due to insufficient information provided by almost all the studies. In terms of other criteria, the majority were classified as having a "low risk" of bias, as they utilized well-defined methodologies and thorough reporting. Only three studies were deemed to have a moderate risk, while four studies were identified as exhibiting a high risk (Figure 3A and C).

In our systematic review of studies using animal models, none were identified as having a high risk of bias according to the SYRCLE tool. However, most studies lacked adequate information to evaluate critical aspects such as “allocation concealment,” “random housing,”, “blinding (performance bias)”, “random outcome assessment”, and “blinding (detection bias)”. Despite this, a significant proportion of the studies were classified as “low risk” in several areas, including “sequence generation”, “baseline characteristics of the animals”, “incomplete outcome data”, and “selective outcome reporting”. Overall, these studies provided limited information regarding animal selection strategies, performance methods, and whether researchers manipulating the animals were aware of group assignments (Figure 3B and D).

### Nomenclature and cancer type distribution in EndMT research

We first examined the nomenclature used in each selected study to refer to the EndMT process, as the terminology employed in databases can significantly influence the studies analyzed. We observed a variety of terms referring to the same process (see Table 4), with “endothelial-to-mesenchymal transition (EndMT)” being the most common. This nomenclature was adopted for the present article. Conversely, other terms such as “endothelial mesenchymal transition” 30 and “endothelial-mesenchymal transformation” 31 were less common. Notably, one study on breast cancer used the term “endothelial to mesenchymal transition” but employed the abbreviation “EMT” 32, highlighting a potential overlap between the concepts of EndMT and “epithelial-to-mesenchymal transition (EMT)”. Indeed, we identified a potential source of confusion in the literature, as some studies initially seemed to meet our inclusion criteria but were ultimately excluded because they focused on the EMT phenomenon in cancerous cells, despite using the term "endothelial-to-mesenchymal transition." 33,34. These findings underscore the need for careful analysis to distinguish between EndMT and EMT processes.

Next, we assessed the cancer types addressed in each paper. As shown in Table 5, the EndMT process is most commonly studied in breast cancer. For example, one study found that tumour-associated endothelial cells (TECs) in breast cancer undergoing EndMT downregulated the endothelial-like marker PECAM1 and demonstrated enhanced migration and extracellular matrix remodeling capabilities compared to normal endothelial cells 35.

In contrast, we discovered limited information on the induction of EndMT in certain tumours, such as esophageal adenocarcinoma 36 and nasopharyngeal carcinomas 37. Notably, we identified no experimental studies focusing on bladder, prostate or gastric cancer, indicating promising avenues for future research. For instance, gastric cancer is the fourth leading cause of cancer-related death worldwide 38, metastatic prostate cancer remains incurable 39, and secondary, highly aggressive bladder cancers frequently develop after radiotherapy for prostate tumours 40, along with the induction of EndMT by radiotherapy 41.

It's worth mentioning that the same article examined EndMT across multiple cancer types, for example melanoma, breast and lung cancer, revealing a correlation between the induction of EndMT and a poor disease prognosis 42. Furthermore, most selected studies evaluated EndMT in blood vessel-related endothelial cells, with only four focusing in lymphatic endothelial cells (Table 6). Of these, two studies related to cervical 43 and oral squamous cell carcinoma 44 found a positive correlation between the development of EndMT in lymphatic endothelial cells and an increased frequency of lymph node metastases.

### Direct induction of EndMT during tumour progression and alterations in tumour biology caused by the EndMT process (Feedback Mechanisms)

Table 6 illustrates that the majority of studies (n=42) reported the induction of EndMT by the tumour mass. For instance, one article showed that hepatocellular carcinoma cells induced EndMT in human umbilical vein endothelial cells (HUVECs) through the secretion of protein dickkopf-1, an antagonist of Wnt molecular pathway (dickkopf-1), which was characterized by increased migration, invasion, tube formation, and angiogenic capabilities 45. Conversely, a smaller number of publications (n=31) reported feedback effects on tumour progression arising from the triggering of EndMT process itself. One study indicated that EndMT enhanced tumour growth in mouse models of melanoma 46. Notably, some studies addressed both the direct induction of EndMT and its feedback effects (alterations in tumour biology, such as increased tumour growth, metastasis, or enhanced tumour-associated fibrosis, triggered by EndMT itself) simultaneously, for instance in some studies related to pancreatic cancer 47-49.

We also observed a potential mechanistic relationship between EMT in tumour cells and EndMT in endothelial cells. Several articles suggested that malignant cells (e.g., oral squamous cell carcinoma, breast cancer, colon cancer) exhibiting EMT and higher invasive capacities 50-52 may be more effective at inducing EndMT in endothelial cells than epithelial-like cancerous cells. Furthermore, endothelial cells undergoing EndMT induced EMT in their surrounding tumour cells of oral squamous cell carcinoma 53 and breast cancer 54.

Supporting this notion, a study on glioblastoma revealed that chemoresistant tumour cells, known for their ability to undergo EMT, were more effective at triggering EndMT compared to chemosensitive cells. The same work documented that endothelial cells (hCMEC line) undergoing EndMT also contributed to tumour chemoresistance 55. Interestingly, we found no articles addressing the potential of cancer-stem cells (CSCs) to induce EndMT, presenting an intriguing research opportunity given the established links between chemoresistance, EMT, and the CSC phenotype 56. However, we did identify two studies, focused on lung 57 and breast cancer 32, indicating that tumour spheroid structures, often associated with CSCs, induced EndMT more effectively than standard monolayer cultures, although the presence of CSCs within these 3D structures was not clearly defined. Equally, some authors noted that endothelial cells undergoing EndMT (with downregulation of CD31, VE-Cadherin, Tie1, Tie2 endothelial-like factors and induction of αSMA, fibronectin, TWIST1, SNAIL mesenchymal-like ones) could promote proliferation, invasion, migration, and stemness in colorectal cancer cells 58.

Regarding the interactions between endothelial cells and other stromal cells within the TME, it is well documented that EndMT facilitates the transformation of endothelial cells into CAFs. For instance, invasive colon cancer cells have been proved to induce the transdifferentiation of endothelial cells (HMEC-1 line) into CAFs, with a more elongated morphology and increased migratory abilities, through the EndMT process 59. Despite this, we identified only a limited number of articles (n=4) focusing on the induction of EndMT by stromal cells. One study in cervical squamous cell carcinoma showed that the activation of the EndMT process by CAFs, through the secretion of PAI-1, enhanced the proliferation, migration, and permeability of endothelial cells. In fact, the cytokine PAI-1 binds to its receptor LRP1 on endothelial cells, thereby activating the ERK/AKT signaling axis, which enhances the proliferation, migration, and permeability of endothelial cells 43. Additionally, EndMT induction has been shown to trigger the recruitment, infiltration, and M2 polarization of macrophages 60. Correspondingly, Ji and colleagues demonstrated that TAMs, conditioned by ovarian cancer cells, promoted EndMT and enhanced tube formation of HUVECs 61. Similarly, another study found that mast cells activated by extracellular vesicles derived from non-small cell lung cancer cells could also induce EndMT along with the migration and tube formation capabilities of HUVECs 62. These limited, yet significant, findings underscore the need for further exploration of the interactions between endothelial cells undergoing EndMT and tumour-related stromal cells.

Finally, we identified a limited number of studies (n=8) documenting the induction of EndMT by microenvironmental conditions closely linked to tumour biology. For instance, one study on breast cancer reported that 27-hydroxycholesterol activated the EndMT process in HMEC1 and HUVECs endothelial cells through their direct stimulation with the aforementioned molecule which promotes the activation/phosphorylation of STAT3 51, suggesting a potential connection between obesity and cancer development. Additionally, a study on pancreatic cancer, revealed that an acidic microenvironment, a well-known hallmark of solid tumours, promotes EndMT along with increased migration, invasion, and permeability of endothelial cells 63.

Several studies have also shown that altered blood flow and fluid shear stress, frequently observed within the dense TME, may play a crucial role in triggering EndMT 64-66. Similarly, hypoxia condition, another hallmark of solid tumours, has been demonstrated to enhance the ability of TGFβ1 (a well-known inducer of EndMT) to trigger the EndMT process characterized by the downregulation of CD31 and VE-Cadherin 67. Furthermore, Smeda and colleagues found that advanced age increased the induction of EndMT triggered by tumour cells of breast cancer 68.

Diabetes mellitus, a risk factor for cancer, is associated with elevated glucose levels. Notably, a study on colon cancer showed that high glucose levels promoted EndMT characterized by an upregulation of vimentin, SNAIL and αSMA 69.

**Figure 4:**
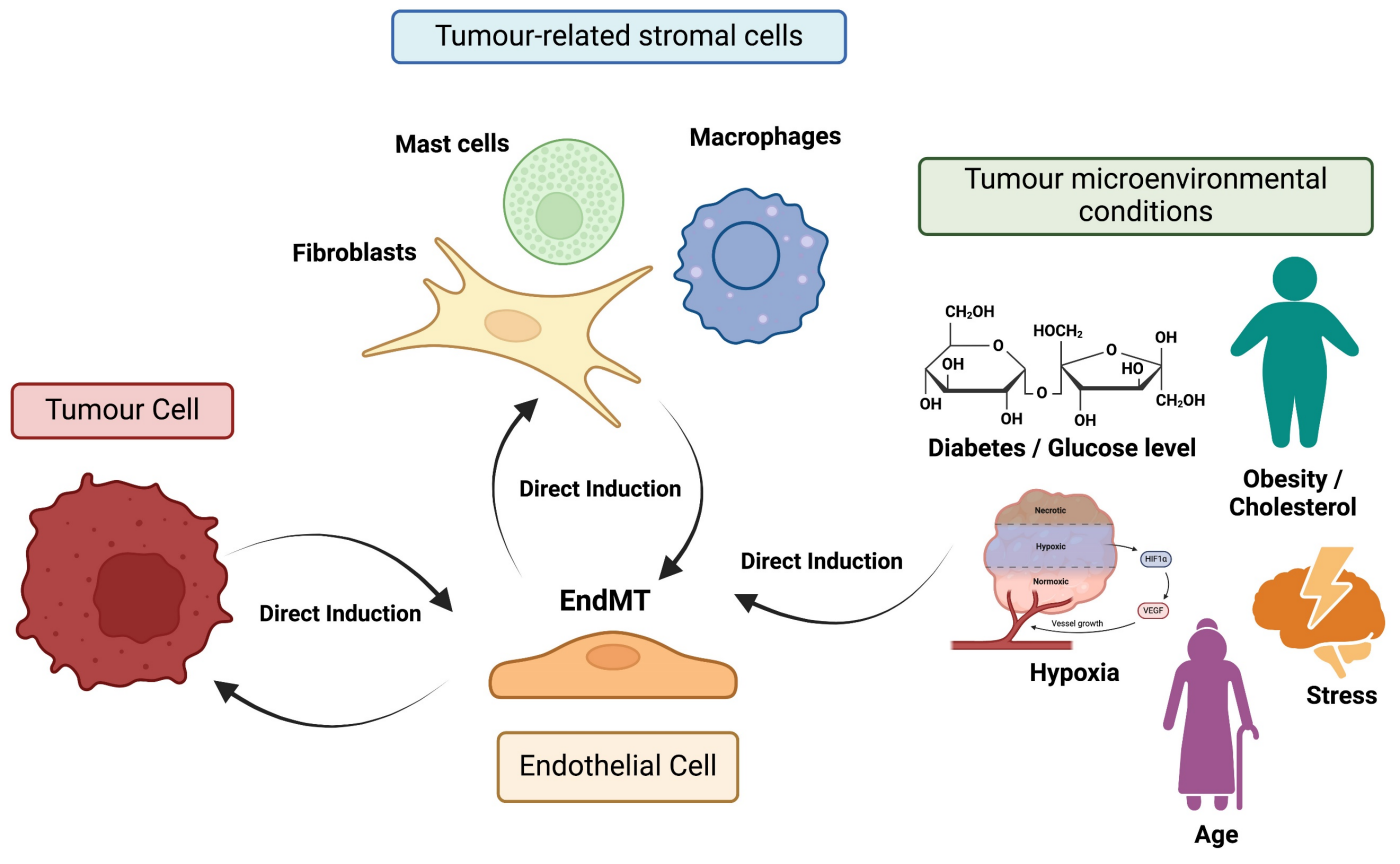
** Direct induction of EndMT and feedback mechanisms.** Factors and signals received by endothelial cells from tumour cells, tumour-associated stromal cells (e.g., macrophages), and various microenvironmental conditions (e.g., hypoxia) can promote or enhance signaling pathways involved in the induction of EndMT. Furthermore, endothelial cells undergoing EndMT can provide feedback to these surrounding cells, significantly contributing to tumour progression and metastasis.

Figure 4 offers a graphical summary of the relationships between endothelial cells undergoing EndMT, tumour cells, stromal cells and tumour hallmarks.

### Experimental approaches in EndMT research: determining the molecular pathways involved

Table 7 shows a classification of the various experimental methods employed in the selected studies. The three primary approaches included *in vitro* studies, using cell line models (n=52), *in vivo* experiments with animal models (n=31), and analyses of human patient samples (n=18). One study that focused on hepatocellular carcinoma, examined EndMT using all three experimental approaches in conjunction 70. Furthermore, it is worth noting that the culture of 3D cellular structures allows for a more precise characterization of EndMT, as these structures better mimic the architecture and biology of living tissues compared to monolayer-based cell cultures. In this context, Kim *et al.* developed a microfluidic device that mimics the liver microenvironment to investigate the formation of the premetastatic niche influenced by extracellular vesicles from breast cancer cells, emphasizing the significance of EndMT in this process 71. Patient-derived xenografts (PDXs) have also been used to investigate EndMT. For instance, one study utilized PDX models of pancreatic ductal adenocarcinoma to define a reversible EndMT, which significantly enhanced tumour-associated desmoplasia 72.

In our study, we investigated the types of endothelial cells utilized in the *in vitro* experiments designed to evaluate EndMT. These endothelial cell models are chosen for their relevance to specific organs or their ability to recapitulate key features of the TME.

Our findings indicate that a range of endothelial cell lines were employed, as detailed in Table 8. The most frequently used cell line was human umbilical vein endothelial cells (HUVEC), with 29 studies utilizing this model; however, a considerable number of articles (n=25) were based on alternative endothelial cell lines. For instance, human brain microvascular endothelial cells (HBMEC) were employed to elucidate the role of EndMT, increasing vascular permeability, in the formation of brain metastases induced by non-small cell lung cancer cells 73. Similarly, induced pluripotent stem cell-derived brain microvascular endothelial cells (IBMECs) were utilized to investigate the mechanisms by which breast cancer cells traverse the blood-brain barrier via EndMT induction with an enhancement of endothelial barrier permeability 74. Additionally, human lung microvascular endothelial cells (HMVEC-L) were studied to further understand the induction of EndMT by non-small cell lung carcinoma cells, revealing a correlation between such molecular process and the development of metastases 75. Several studies utilized multiple endothelial cell lines concurrently, such as human microvascular endothelial cells (HMEC1) and bovine aortic endothelial cells (BAEC) in a research concerning pancreatic cancer 76. Furthermore, mouse endothelial cells, including primary rat brain endothelial cells (RBEC), were also employed to assess the impact of EndMT induced by melanoma and breast cancer cells on the permeability of endothelial monolayers, facilitating tumour cell transendothelial migration 25. Moreover, several studies isolated and directly analyzed tumour-associated endothelial cells (TECs) from mouse xenografted tumour tissues 77 and human patient tumour biopsies 78 to characterize the EndMT process. This variety of approaches underscores the importance of using multiple endothelial cell lines to gain a comprehensive understanding of EndMT in different tumour contexts.

We further analyzed the methodologies used to investigate the molecular markers and functional properties of endothelial cells undergoing EndMT, focusing primarily on direct co-cultures of endothelial cells with tumour cells 79 and indirect cultures using conditioned medium from tumour cells 24. Notably, one study demonstrated that direct cell-cell contact between cancerous and endothelial cells may be essential for inducing EndMT, as conditioned medium from breast cancer cells alone did not initiate this phenotypic transition 80.

Next, our review identified several experimental approaches for measuring specific molecular and functional characteristics of EndMT. Biomarker expression levels were predominantly assessed using quantitative PCR (qPCR), immunofluorescence detection, western blotting, and flow cytometry. Flow cytometry, in particular, proved to be effective for accurately detecting changes in various cell subpopulations based on the expression of specific markers (e.g., αSMA), even when other methods like qPCR or western blotting did not reveal significant alterations 81. Additionally, single-cell RNA sequencing was also employed to assess the heterogeneity of different cell subpopulations undergoing EndMT concerning their whole transcriptional profiles 79. Table 9 highlights the diverse endothelial-like factors that were downregulated during the EndMT process, alongside the mesenchymal-like factors that were upregulated, as identified in studies with multiple instances (n>1). This heterogeneity underscores the importance of incorporating a comprehensive array of biomarkers in the design of future research studies.

Additionally, we observed a wide range of experimental assays assessing various functional properties altered during EndMT (Table 10). Notably, the results in this area showed considerable heterogeneity between different studies. As an example, some studies reported an enhanced proliferation capacity of endothelial cells during EndMT 82, while others indicated no changes 62 or even a reduction in proliferation rates 83. These discrepancies in biomarker expression and functional properties may stem from several factors, including the inherent heterogeneity of the EndMT process, differing microenvironmental conditions, variations in tumour and stromal cell types, distinct experimental setups, or the specific endothelial cell types under investigation.

Finally, we identified several molecular factors that can induce EndMT, as summarized in Table 11. A substantial number of studies emphasized the role of TGFβ ligands (e.g., TGFβ1 and TGFβ2) secreted by own cancerous cells and/or stromal cells in triggering the EndMT process (n=25). Some publications utilized TGFβ1-induced EndMT as a positive control for comparing the EndMT initiated by other cell types, such as breast cancer or melanoma cells 84,85. However, it is important to note that TGFβ1 and TGFβ2 should not always be regarded as interchangeable in the context of EndMT induction. For instance, a study focusing on esophageal and colon cancer demonstrated that IL-1β and TGFβ2 (but not TGFβ1) could promote EndMT in human endothelial microvascular cells (HEMEC line) and human intestinal microvascular endothelial cells (HIMEC line) 36. Additionally, some studies reported that EndMT can be induced through the combined action of multiple factors, such as TGFβ2 and IL-1β within the melanoma context 81.

**Figure 5:**
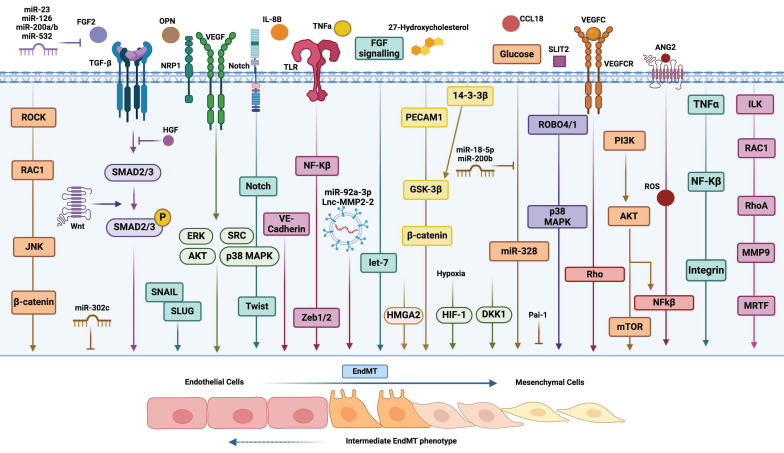
** Inducers and molecular pathways of EndMT.** A diagrammatic representation illustrating the induction of EndMT by various secreted factors and distinct molecular pathways. This figure highlights the complex interactions and signaling mechanisms involved in the transition of endothelial cells to a mesenchymal phenotype.

The significant heterogeneity of the molecular pathways underlying EndMT in endothelial cells is summarized in Table 12. For example, in pancreatic cancer, the upregulation of L1CAM in tumour-associated endothelial cells (TECs) enhanced IL-6 expression, activating the JAK/STAT3 signaling pathway, which in turn triggered EndMT associated with tumour progression and metastasis 86. Interestingly, Akatsu *et al.* identified several axes of heterogeneity, demonstrating that the TGFβ2/MRTF-A (myocardin-related transcription factor A)/SRF (serum response factor) axis drives a myofibroblast-like transition in endothelial cells (End-myoT), while the FGF2/ELK1 (ETS-like transcription factor 1)/SRF axis leads to a non-myofibroblast-like EndMT (End-N-myoT) 87. Specifically, this study showed that FGF2 antagonizes TGFβ2, promoting a non-myofibroblastic phenotype in endothelial cells through the activation of ELK1. This occurs because MRTF-A and ELK1 compete for binding to the same serum response factor. Moreover, partial or incomplete EndMT induced by breast cancer cells was characterized by the simultaneous expression of endothelial markers such as CD31 and VE-Cadherin, alongside mesenchymal markers including αSMA, FSP1, vimentin, and fibronectin 80. It is important to note that this study does not clarify the reasons behind the development of partial, rather than complete, EndMT. This could be attributed to various factors, such as short co-culture durations or the activation of specific molecular axes, as the expression of endothelial and mesenchymal markers may not be regulated by the same molecular factors or pathways. Some studies reported reversible EndMT requiring a constitutive induction by tumour cells 72, while others indicated an irreversible transition 24. This molecular heterogeneity is illustrated in Figure 5.

### Meta-analysis of EndMT-related biomarkers: exploring expression heterogeneity and correlations with patient prognosis

**Figure 6:**
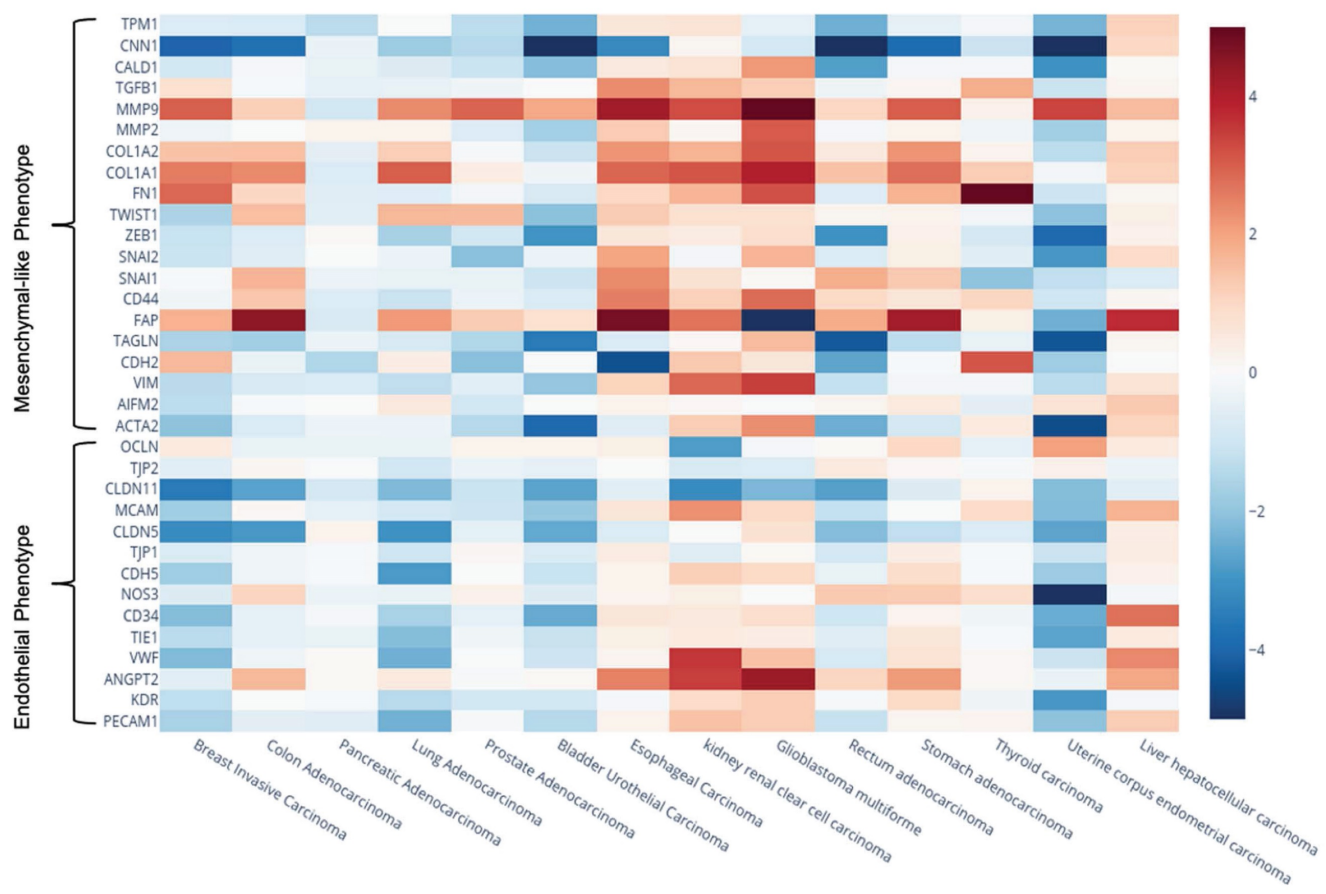
** Gene expression heatmap showing the Log₂(Fold Change) of genes of interest across different cancer types.** Expression data (TPM) were retrieved from the UALCAN database. The color scale was adjusted between values of -5 and 5 to enhance the visual representation of expression changes.

We conducted a meta-analysis on the expression levels of previously identified EndMT-related markers, focusing on both endothelial-like and mesenchymal-like markers. This analysis aimed to substantiate the expression heterogeneity of EndMT-related biomarkers across various tumours types, utilizing a larger sample size from the UALCAM database. We compared tumour tissues to their corresponding healthy tissues (Figure 6, Supplementary Table S1). Notably, we included three additional endothelial-like genes associated with cell-cell junctions, MCAM, CLDN11, and TJP2, that are known to be downregulated during the EndMT process. These additional markers were identified in only one article within our systematic review, reporting the downregulation of MCAM (CD146) in glioblastoma 24, CLDN11 (claudin 11) in colon cancer 82, and TJP2 in melanoma and breast cancer 25.

Our findings revealed significant variability in expression levels, both within individual tumour types and across different solid tumours. For instance, in renal clear cell carcinoma endothelial-like genes such as ANGPT2 and VWF were notably upregulated, whereas OCLN levels were significantly decreased. Similarly, VWF and CD34 showed significant upregulation in hepatocellular carcinoma, contrasting with their downregulation in breast and lung adenocarcinomas. The mesenchymal-like gene VIM was found to be downregulated in bladder urothelial carcinoma but upregulated in renal clear cell carcinoma and glioblastoma. Consistent with previous studies 88, we observed significant upregulation of FAPα and matrix metalloproteinase 9 (MMP9) across the majority of cancer types analyzed, highlighting their relevance in tumour biology, as they have been associated to tumour progression and/or metastasis.

Given that mesenchymal-like factors can be influenced by both EndMT in endothelial cells and EMT in tumour cells, we concentrated on alterations in endothelial-like markers as more specific indicators of active EndMT process. Table 13 presents endothelial-like biomarkers that were downregulated with a fold change of less than -1.5 across various tumour types compared to healthy tissues, all exhibiting high statistical significance (p < 0.05). Note that we used a 1.5-fold cut-off, as it allowed us to focus on genes with statistically significant fold changes, in line with previous studies 89-91. The results suggest that EndMT may be more actively occurring in breast invasive carcinoma, lung adenocarcinoma, bladder urothelial carcinoma, and uterine corpus endometrial carcinoma, as indicated by significant downregulation of numerous endothelial-like markers.

**Figure 7:**
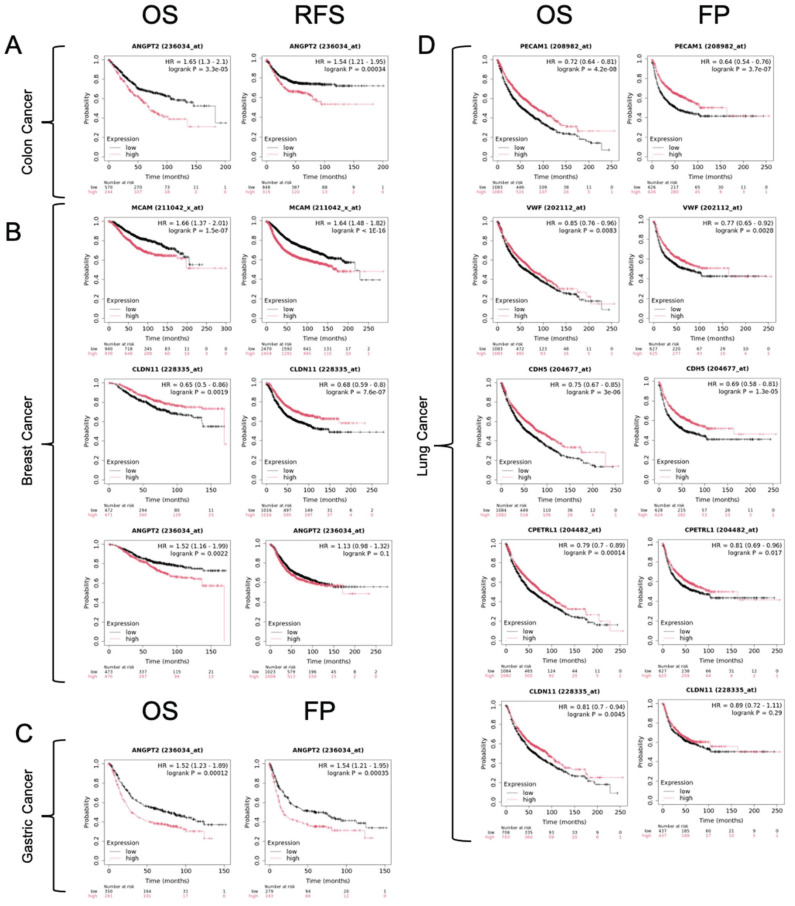
** Kaplan-Meier survival curves illustrating the impact of gene expression levels on patient survival outcomes**. Panel (A) shows overall survival (OS) and relapse-free survival (RFS) for colon cancer, and panel (B) shows the same outcomes for breast cancer. Panel (C) displays OS and first progression (FP) for gastric cancer, while panel (D) shows the same for lung cancer. The plots compare high (red line) vs. low (black line) expression. Each panel includes the hazard ratio (HR) and the associated log-rank p-value, indicating the statistical significance of expression differences on survival.

We also analyzed correlations between the downregulation of these endothelial-like factors and patient prognosis using the KM/plotter database (Table 14, Figure 7). Notably, reduced expression of MCAM and CLDN11 in breast cancer correlated with poor overall survival and relapse-free survival. Furthermore, downregulation of PECAM1, VWF, CDH5, CLDN5, and CLDN11 was associated with poor overall survival and progression in lung cancer, which may emphasize a critical role of EndMT in these malignancies. Interestingly, our meta-analysis also indicated a trend of ANGPT2 upregulation across several cancer types, including colon, esophageal, glioblastoma, stomach, hepatocellular, and renal clear cell carcinomas, suggesting its involvement in tumour progression. We found correlations between increased ANGPT2 expression and poor overall survival, relapse-free survival, and first progression in breast, colon, and gastric cancers (Table 14, Figure 7).

Additionally, in contrast to breast invasive carcinoma and lung adenocarcinoma, several mesenchymal-like markers were also downregulated in bladder urothelial carcinoma and uterine corpus endometrial carcinoma apart from the previously mentioned downregulation of endothelial-like factors. This may suggest the predominance of a partial/incomplete EndMT or the possible upregulation of different mesenchymal-like genes during EndMT.

## Discussion

In this study, we aimed to elucidate the current "state of the art" regarding EndMT in solid tumours while identifying potential weakness in the experimental evidence. Our analysis indicates that several cancer types, such as esophageal adenocarcinoma, ovarian, gastric, bladder, and prostate cancers, are under-researched in relation to EndMT. Notably, emerging data suggest a significant correlation between EndMT induction and the progression of diffuse gastric cancer, as underscored in an *in silico* study 92. This finding emphasizes the critical need for additional experiments-based research into EndMT in gastric cancer to fully understand its implications for tumour biology and treatment strategies. Prostate cancer, known for its significant fibrosis and strong association between CAFs and tumour progression, metastasis, and therapy resistance 93, also presents a compelling area for further research. Evaluating the contribution of EndMT to the fibrosis and progression of prostate cancer could provide valuable insights. Similarly, breast 94 and pancreatic cancers 95 are known for their fibrotic characteristics. Notably, distinct CAF subtypes with specific and sometimes contradictory roles in tumour progression have been identified even within the same malignancy, such as pancreatic ductal adenocarcinoma 96. Investigating whether EndMT is associated with particular CAF subtypes and their roles in tumour biology merits deeper investigation.

Additionally, we identified a limited number of studies addressing the induction of EndMT in lymphatic endothelial cells and their role in lymph node metastasis. This angle may be especially relevant in solid tumours characterized by significant lymphangiogenesis and lymph node metastases correlating with disease progression, as seen in bladder 97, gastric 98, and esophageal carcinomas 99 as representative examples. The presence of lymph node metastases arises as a postoperative predictive factor of cancer recurrence, poor overall survival and advanced tumour progression stage in many tumour types (i.e. pancreatic or colon cancer) 100,101. Interestingly, the loss of lymphatic endothelial barrier tight junctions/integrity and increasing lymphatic vessel permeability (potentially disturbed during EndMT) has commonly been considered as an event leading to lymph node metastasis, thereby facilitating tumour cell intravasation 102. Additionally, mesenchymal-like lymphatic endothelial cells contribute to extracellular matrix remodelling, creating a pro-metastatic niche within lymph nodes that supports tumour cell colonization 103. While our focus has been primarily on solid malignancies, it's important to acknowledge that EndMT has also been correlated with cancer progression in liquid malignancies, such as diffuse large B-cell lymphoma 104.

The relevance of the functional interplay between endothelial cells undergoing EndMT and tumour or stromal cells has also been explored in this review. Most selected studies reported tumour cells as inducers of EndMT. However, the potential for CSCs to trigger EndMT in stromal TECs remains underexplored. This is particularly intriguing, as CSCs are thought to modify stromal cells more efficiently toward a pro-tumour phenotype (myofibroblast-like CAFs and M2-polarized TAMs) compared to non-stem cancer cells 105. We identified only one study that demonstrated that CAFs could induce EndMT 43. This finding is significant and highlights the need for a deeper understanding of the mechanisms by which CAFs influence endothelial cell behavior. Moreover, the parallels between endothelial cells undergoing EndMT and CAFs behavior suggests that these processes are not isolated but rather interconnected within the broader context of tumour biology. Indeed, CAFs have the capacity to "educate" other stromal cells like normal fibroblasts into a pro-inflammatory phenotype, which can exacerbate tumourigenesis 106. Our findings also suggest a positive correlation between EndMT and M2 polarization of TAMs, similar to the exposed ability of CAFs to promote M2 polarization, for instance, of glioma TAMs 107. Understanding how CAFs mediate these transitions, including EndMT process, may provide insights into potential strategies for targeting the tumour stroma to improve treatment outcomes.

However, we did not identify any studies examining the influence of EndMT on the infiltration or cytotoxic activity of other types of immune cells, such as T or NK cells. This area is significant, considering that specific CAF subsets have been shown to restrict CD8+ T cell infiltration and tumour cytotoxic activity in head and neck carcinoma 108, and that CAFs can inhibit NK cells cytotoxicity by inducing ferroptosis in gastric cancer 109. Thus, further exploration of the cross-talk between EndMT and immune cells is warranted. Notably, a bioinformatic study indicated strong intercellular communication between CAFs with EndMT profiles and TAMs in breast, gastric, and colorectal cancers, correlating this interaction with poor prognosis according to TCGA data 110. Furthermore, several studies have established links between EndMT and common risk factors for cancer, such as obesity and high glucose levels, suggesting that EndMT could contribute not only to tumour progression but also to tumourigenesis.

Our findings indicate a notable trend characterized by the downregulation of VE-Cadherin, an endothelial-specific marker, coupled with the upregulation of αSMA, a mesenchymal marker, and activation of the TGFβ pathway during EndMT. However, our analysis also revealed significant heterogeneity at both the molecular and functional levels across different studies. This variability can be attributed to a range of factors. One major contributor is the diversity of endothelial cell lines and types utilized in these investigations. Endothelial cells are not a homogeneous group; rather, they exhibit substantial differences depending on their anatomical location within the body. This inherent variability has been observed at the levels of cell morphology, functions, expression profiles, antigen composition and response to TGFβ2 111. Moreover, one study on melanoma revealed a subpopulation of TECs that possess antitumour properties, functioning through the L-PDGS/PGD2/DP1 (lipocalin-type prostaglandin)/PGD2/DP1 signaling axis. Specifically, this subset of endothelial cells can express the enzyme L-PGDS which catalyzes the formation of PGD2 (prostaglandin D2) that binds to its receptor DP1 inhibiting the EndMT process along with tumour growth 46. This discovery reinforces the significant phenotypic heterogeneity within endothelial cell populations, which mirrors the variability observed among different subtypes of CAFs found within the same TME.

To obtain more precise and meaningful experimental results, it is crucial to utilize tissue-specific endothelial cell lines or directly isolate and analyze TECs from patient samples. The use of techniques such as immunomagnetic column-based isolation could effectively minimize contamination from CAFs and tumour cells, thereby enhancing the reliability of the data collected from isolated TECs 112. This targeted approach enables researchers to capture the unique characteristics and behaviors of TECs, fostering a deeper understanding of their roles within the TME and their potential as therapeutic targets.

The characteristics of EndMT may also be heavily influenced by tumour types and specific cancer cell lines, reflecting variable molecular inductors and different molecular axes. While our data suggest a tendency to promote control EndMT in laboratory settings through TGFβ exposure, some studies have noted significant differences in the effects of TGFβ isoforms on EndMT induction, with TGFβ2 being the more effective isoform compared to TGFβ1 113. Furthermore, distinct responses to TGFβ2 exist between healthy endothelial cells and TECs from various tissues, tumour types, and even subclones within the same culture 114,115. When establishing *in vitro* models of EndMT, key considerations should include the type of endothelial cells used, the specific inducers applied, their dosage, exposure duration, potential interactions between inducers, and microenvironmental factors such as oxygen levels 116. Notably, exosomal TGFβ has been shown to induce EndMT more efficiently than soluble TGFβ, emphasizing the importance of EndMT induction methodology 64.

In addition, the versatility of EndMT should not be underestimated. While much of the research has focused on the CAF-like variant of EndMT in cancer, it is essential to recognize the potential emergence of other mesenchymal-like cell types from this phenotypic transition. One candidate could be pericytes, which also play significant roles in fibrosis and myofibroblast expansion 117. Interestingly, one study identified two distinct variants of EndMT, including the End-N-myoT variant which does not lead to a myofibroblast-like phenotype in endothelial cells 87. Furthermore, the reversibility of the EndMT process could significantly influence tumour progression, analogous to the well-documented MET (mesenchymal-epithelial transition), which is critical for metastatic colonization 118. However, research on mesenchymal-to-endothelial transition (MEndT) in cancer remains limited. For example, only a few studies have suggested that CAFs in pancreatic cancer can transdifferentiate into endothelial cells via MEndT, contributing to tumour angiogenesis and tumour progression 119. Table 15 summarizes the key research gaps identified in this study.

The plasticity inherent in EndMT positions it as a promising therapeutic target in cancer treatment. For instance, inhibiting the TGFβ pathway could simultaneously suppress EndMT and tumour progression. Similar strategies have been proposed, such as blocking TGFβ signaling and the traditional EMT process in glioblastoma-associated pericytes to disrupt tumour angiogenesis 120. Some studies have also identified an anti-cancer compound, like eribulin, that inhibits EndMT triggered by TGFβ stimulation and reduces tumour angiogenesis 121. Similarly, other publication showed that Nudifloside, a derivative of Callicarpa nudiflora used in China as a traditional folk medicine, can reverse the EndMT (through inhibiting Ezrin phosphorylation) induced by TGFβ1 122, thus revealing a potential anti-EndMT drug for cancer therapy. In this line, the EndMT process has also been implicated as a cellular response to standard anti-cancer radiotherapy regimens, potentially linked to tumour regrowth, therapy resistance, metastasis, and the emergence of CSCs 41.

Despite the promising potential of therapies targeting EndMT, the significant heterogeneity of the EndMT process must be carefully considered when developing specific inhibitory drugs to avoid unintended consequences 123. For example, while TGFβ pathways may exhibit tumour-restrictive effects in early stages of cancer, they can promote tumour progression in advanced stages, complicating therapeutic strategies aimed at their blockade 124. Furthermore, anti-EndMT approaches could target tumour vasculature-specific biomarkers to minimize potential side effects in healthy tissues, as the EndMT process has also been observed in normal physiological processes, such as heart valve development 125. Thus, here it is reanimated the significant heterogeneity observed in the expression levels of EndMT-related markers. This variability is evident not only between different tumour types but also within individual malignancies, influenced by various factors and the simultaneous occurrence of multiple molecular processes. Consistent with findings in breast cancer, where the upregulation of endothelial-like genes such as VWF and CD34 correlates with increased angiogenesis, the downregulation of VE-Cadherin has been linked to EndMT 84. In this context, the careful selection of the appropriate EndMT-related biomarker(s) is critical for developing an effective anti-EndMT drug, rather than an anti-angiogenic one, which may be associated with frequent inefficiency and adverse effects in cancer therapy 126. Our review also indicates a markedly high activation of the EndMT process in lung adenocarcinoma. Notably, we establish, at the best of our knowledge, for the first time a statistical correlation between the downregulation of CLDN11 and poor prognosis in this cancer type. Interestingly, previous research has documented high CLDN11 expression in tumour cells from human lung squamous cell carcinoma 127, reinforcing the idea that the downregulation of CLDN11 identified in our analysis may predominantly arise from EndMT in endothelial cells. This underscores the complex interplay between endothelial plasticity and tumour biology, highlighting the need for further investigations into these relationships.

Interestingly, our investigation highlighted ANGPT2 overexpression in several tumour types, particularly esophageal carcinoma, which may warrant future studies to evaluate its correlation with patient prognosis, given that ANGPT2 has previously been validated as a consistent biomarker in esophageal squamous cell carcinoma 1.

Nevertheless, it is important to acknowledge some limitations in our systematic review. Specifically, a limited number of studies on certain tumour types (e.g., ovarian or nasopharyngeal carcinoma) were included, which prevents us from drawing robust, cancer-specific conclusions until further research is conducted. Regarding the meta-analysis, the expression levels of specific genes can be influenced by a variety of factors in clinical settings, in addition to the previously noted high heterogeneity of EndMT. For instance, the stage of tumour progression at the time of sample collection can significantly impact the interpretation of biomarker significance. Early-stage tumours may exhibit distinct molecular profiles compared to advanced-stage malignancies 128. Furthermore, individual genetic and epigenetic profiles play a crucial role in tumour biology and response to treatment. Variability in genetic mutations, polymorphisms, and epigenetic modifications among patients can lead to differences in expression profiles (including those related to EndMT) 129, complicating the establishment of universally applicable conclusions. These factors underscore the need for a more nuanced understanding of how molecular alterations operate in diverse patient populations.

Additionally, specific anticancer regimens, doses, and administration formats can influence tumour cell expression profiles and their interactions with the surrounding stroma 130. Consequently, patients who have undergone prior treatments, such as chemotherapy, radiotherapy, targeted therapies, or immunotherapy, may exhibit changes in biomarker expression related to EndMT. These changes could potentially confound meta-analysis results and lead to inconsistencies across studies. Given these complexities, it is important to approach the statistical findings of this meta-analysis with caution. Only through rigorous experimental validation can we fully understand the role of EndMT-related biomarkers in cancer progression and their potential as therapeutic targets.

## Conclusions

This study highlights the current understanding of EndMT in solid tumours, emphasizing the substantial areas where further research is needed, particularly in cancers such as esophageal adenocarcinoma, ovarian, gastric, bladder, and prostate cancer, and regarding the implication of EndMT in lymph node metastases.

Our findings also indicate that EndMT could intricately be linked to CAFs and TAMs, suggesting a complex interplay between endothelial cells and TME components. Notably, the influence of CSCs on inducing EndMT in stromal endothelial cells remains underexplored, presenting a promising avenue for future research.

Moreover, the variability in EndMT markers expression and phenotypic variations in endothelial cells across different tumour types and within the same tumour suggests that the process is influenced by a multitude of factors, including cancer type, endothelial cell type, microenvironmental conditions, and specific molecular inducers. This heterogeneity may complicate the development of targeted therapies aimed at inhibiting EndMT. The reversibility and plasticity of EndMT underscore its potential as a therapeutic target, especially through the modulation of pathways like TGFβ. However, the dual nature of TGFβ signaling across different cancer stages requires caution in therapeutic strategies. Understanding EndMT as a multistep process, rather than a simple biphasic transition, allows for the possibility of detecting partial or "incomplete" EndMT under specific conditions.

Overall, our meta-analysis not only confirms the high variability in EndMT-related markers but also establishes a possible statistical correlation between EndMT and lung cancer, remarking the downregulation of CLDN11 and poor prognosis, alongside the upregulation of ANGPT2 with its possible role as a potential biomarker in esophageal carcinoma. These insights call for continued research to deepen our understanding of EndMT in cancer biology and its implications for treatment strategies.

## Supplementary Material

Supplementary tables.

## Figures and Tables

**Table 1:** Inclusion and exclusion criteria for the searching process.

Category	Inclusion criteria	Exclusion criteria
Article type	In vitro, *in vivo* and/or patient samples analysis studies evaluating the EndMT process in the context of solid malignancies.	Systematic reviews, meta-analyses, in silico studies, reviews, comments/notes, book chapters, thesis, conference papers/abstracts, editorial letters, clinical trials, observational studies and randomized control trials. Articles with full text not available
Experimental context	Articles evaluating the induction/influence of EndMT on solid tumour biology. Articles evaluating the induction of EndMT by tumour-related hallmarks (i.e. hypoxia, acidic microenvironment)	Articles focusing on physiological development or on pathologies different from cancer disease (i.e. atherosclerosis). Articles about liquid malignancies and tumours derived from the own endothelium (i.e. Kaposi's sarcoma). Studies analysing EndMT conditioned by a particular anti-cancer treatment/drug.
Publication date	From inception until June 2024	Articles published after June 2024
Language	Publications in English language	Different from English language.

**Table 2:** Patient distribution by cancer type (Tumour vs. Control)

Cancer Type	Control (n)	Tumour (n)
Breast Invasive Carcinoma	114	1097
Colon Adenocarcinoma	41	286
Pancreatic Adenocarcinoma	4	178
Lung Adenocarcinoma	59	515
Prostate Adenocarcinoma	52	497
Bladder Urothelial Carcinoma	19	408
Esophageal Carcinoma	11	184
Kidney Renal Clear Cell Carcinoma	11	184
Glioblastoma Multiforme	5	156
Rectum Adenocarcinoma	10	166
Stomach Adenocarcinoma	34	415
Thyroid Carcinoma	34	415
Uterine Corpus Endometrial Carcinoma	35	546
Liver Hepatocellular Carcinoma	34	415

**Table 3:** Selected genes

Gene symbol	Affymetrix ID
ANGPT2	236034_at
VWF	202112_at
CDH5	204677_at
PECAM1	208982_at
CLDN11	228335_at

**Table 4:** Different EndMT nomenclatures

	Nomenclature/abbreviature	Number of articles
Expression	Endothelial mesenchymal transition	2
	Endothelial-mesenchymal transition	16
	Endothelial to mesenchymal transition	6
	Endothelial-to-mesenchymal transition	27
	Endothelial-mesenchymal transformation	1
	Endothelial to mesenchymal transformation	2
Abbreviations	EMT	1
	EnMT	1
	EndMT	40
	EndoMT	11
	Endo-MT	1

**Table 5:** Cancer and endothelium types

Cancer/endothelium type	Number of articles
Breast cancer	16
Pancreatic cancer	9
Melanoma	8
Colon cancer	7
Lung cancer	6
Oral squamous cell carcinoma	4
Liver cancer	2
Cervical cancer	2
Glioblastoma	2
Ovarian cancer	1
Esophageal adenocarcinoma	1
Nasopharyngeal carcinoma	1
Blood vessel endothelial cells	50
Lymphatic endothelial cells	4

**Table 6:** Direct induction of EndMT and feedbacks.

			Number of articles
Direct induction of EndMT by	Tumour cells		42
	Tumour-related stromal cells	Cancer-associated fibroblasts	1
		Tumour-associated macrophages	2
		Mast cells	1
	Tumour microenvironmental conditions	27-hydroxycholesterol	2
		Flow shear stress/altered flow	3
		Hypoxia	1
		Older age	1
		Diabetes/high glucose levels	1
Feedback effects derived from EndMT on tumour cells			31

**Table 7:** EndMT study stages

Experimental stage	Number of articles
Total in vitro	52
In vitro 3D structures	8
Total *in vivo*	32
Use of PDXs	1
Human patient samples analysis	18

**Table 8:** Endothelial cell type

Endothelial cell type	Endothelial cell line	Number of articles
Human endothelial cells lines	BAECS (primary bovine aortic endothelial cell)	2
	EAhy926	1
	ECRF24 (human immortalized endothelial cell)	2
	HBCA.MEC (breast cancer associated endothelial cells)	1
	HBH.MEC (healthy breast endothelial cells)	1
	HBMEC (human brain microvascular endothelial cell)	2
	HCMEC (human cardiac microvascular endothelial cell)	1
	HDLEC (human dermal lymphatic endothelial cells)	1
	HEMEC (human esophageal microvasculature endothelial cell)	1
	HIMEC (human intestinal microvasculature endothelial cell)	1
	HMEC1 (human microvascular endothelial cell)	5
	HMVEC-L (human lung microvascular endothelial cell)	1
	HPMEC (human pulmonary microvascular endothelial cell)	1
	HUAEC (human umbilical artery endothelial cell)	2
	HUVECs	29
	IBMEC (induced pluripotent stem cell-derived brain-specific microvascular endothelial-like cells)	1
	LECs (telomerase-immortalized human lymphendothelial cell)	1
Mouse endothelial cells	LSEC (immortalized human liver sinusoid endothelial cell)	1
	3B-11 (mouse immortalized endothelial cell)	1
	LUEC	1
	MLEC (primary mouse lung endothelial cell)	1
	Mouse hepatic endothelial cell	1
	MS1 (mouse pancreatic microvascular endothelial cell)	3
	RBEC (primary rat brain endothelial cell)	1
Isolated/analysed tumour-related endothelial cells (TECs) from tumour tissues	Total TECs	12
	TLEC (tumour lymphatic endothelial cell)	1

**Table 9:** EndMT-related biomarkers

Phenotype	Context	Biomarker	Number of articles
Loss of endothelial phenotype	Downregulation of endothelial-like biomarkers	CD31 (PECAM1)	20
		VEGFR2	8
		Tie2	4
		VWF (Von Willebrand factor)	3
		Tie1	2
		CD34	2
		ENOS (endothelial nitric oxide syntase)	2
	Downregulation of endothelial cell-cell adhesion molecules	VE-Cadherin/CD144 (CDH5 gene)	34
		Zo1 (TJP1 gene)	9
		Claudin5 (CLDN5 gene)	6
		Occludin1	4
Gain of mesenchymal-like phenotype	Upregulation of mesenchymal/stem-like markers	αSMA (ACTA2 gene)	38
		FSP1 (S100A4)	19
		Vimentin	19
		N-Cadherin	15
		SM22α (TAGLN gene)	11
		FAPα	4
		CD44	2
	Upregulation of mesenchymal transcription factors	SNAIL (SNAI1 gene)	15
		Slug (SNAI2 gene)	8
		Zeb1	7
		TWIST1	6
	Upregulation of extracellular matrix molecules/remodeling factors	Fibronectin	11
		Collagen (COL1A1)	5
		Collagen (COL1A2)	5
		Matrix metalloproteinase (MMP2)	4
		Matrix metalloproteinase (MMP9)	2
	Upregulation of mesenchymal-like ligands	TGFβ1	3
	Upregulation of contraction proteins	Caldesmon	2
		Calponin	2
		Tropomyosin	2

**Table 10:** Experimental assays to measure endothelial cells functional properties during EndMT

Experimental level	Functional assay	Functional property altered in endothelial cells by EndMT	Article reference
*In vitro*	Morphological analysis (cytoskeleton staining)	Morphological changes (from cobblestone-like to spindle-like morphology)	37
	Collagen adhesion assay	Adhesion capacity	52
	Sphere formation assay	Angiosphere formation ability	73
	Collagen gel contraction assay	Contractile capacity	80
	F-actin stress fibers formation assay (F-actin stainin)	Actin stress fibers formation	74
	Wound healing	Migration capacity	38
	PDGF-dependent wound healing	Migration capacity	69
	Transwell invasion assay	Migration/invasion capacities	63
	Matrigel 3D invasion assay	Invasion capacity	48
	Matrigel tube formation	In vitro tube formation capacity	77
	Proliferation assay	Proliferation capacity	55
	Monolayer permeability assay (with fluorescent dextran and/or Evans blue)	Monolayer permeability	34
	Gap formation assay	Monolayer permeability, integrity	29
	Calcein-transfer GAP-junction activity assay	Monolayer permeability, integrity	51
	Transendothelial electric resistance assay	Monolayer permeability, integrity	22
*In vivo*	Matrigel plugs *in vivo* tube formation assay	*In vivo* tube formation capacity	78
	chicken chorioallantoic membrane (CAM) assay	*In vivo* angiogenesis capacity	28
	Cornea puncture assay	*In vivo* angiogenesis capacity	39

**Table 11:** EndMT molecular inducers

EndMT inducers	Number of articles
TGFβ (soluble and/or exosomal TGFβ)	25
IL1β	3
CCL18	2
FGF2	2
TNFα	2
OPN (osteopontin)	2
27-hydroxycholesterol	2
L1CAM	1
DKK1	1
Exosomal HMGA2	1
exosomal miR-92a-3p	1
exosomal Lnc-MMP2-2	1
Jag1	1
HGF	1
Pai-1	1
GSK-3β	1
12(S)-hydroxyeicosatetraenoic acid	1
WNT5β	1
VEGFC	1

**Table 12:** EndMT-related molecular axes

Molecular axis	Article reference
Mir-548ac/YB1/SNAIL/miR-548ac	56
TGFβ/SMAD1,5	77
Jag1/Notch	73
c-met/ECS1/MMP14/VE-Cadherin	21
LRP1/ERK1,2/AKT	36
Canonical TGFβ/SMAD2,3 pathway	76
PITPNM3/PI3K/AKT/GSK-3β/SNAIL	49
CCL18/MEK/ERK	28
CXCL8/CXCR2	54
ErG/FLI1/H3K27AC/miR-126	35
L1CAM/IL6/JAK/STaT3	79
HMGA2/SNAIL	34
TGFβ2/MRTF-A/Srf	45
FGF2/ELK1/Srf	80
TGFβ2/PI3K/tubulin-β3/RAC1	52
L-PGDS/PGD2	39
TGFβ/miR-302c/MTDH	63
12(s)-HETE/NF-Kβ/Zeb1	29
SLIT2/ROBO4/ROBO1	70
TGFβ1/SMAD2,3/NRP1	42
NOX1/NOX2/Reactive Oxygen species	74
OPN/integrin alphaVbeta3/PI3K/AKT/mTOR/HIF1a/TCF12/EZH2/VE-Cadherin	51
PECAM1/AKT/GSK3β/β-catenin	62
TGFβ1/PLEK2/SHIP2	68
SEMA4C/ERK1,2	27
Lnc-MMP2-2/miR-1207-5p/EPB41L5	66
TGFβ/Notch/SNAIL/Slug	77
27-hydroxycholesterol/STAT3 pathway	44
27-Hydroxycholesterol/Reactive Oxygen Species/p38/14-3-3β/GSK3β/β-catenin	47
adrenomedullin/RAMP2	78
TGFβ/ILK/RAC1/RhoA/MMP9/MRTF-A/MRTF-B	45
Tie1/ERK1,2,5/AKT/Slug	69
TGFβ2+TNFα/NF-Kβ/integrin aV/activin A/TGFβ/SMAD2,3	46
TNFα/MAPK/Tie1/SNAIL/Slug/Zeb2	65
WNT5b/ROCK/RAC1/JNK/β-catenin/SNAIL/Slug	37
PCP/WNT Pathway/SNAIL/Slug	90
VEGFC/VEGFR2,3/Rho	48

**Table 13:** Summary of endothelial-like markers downregulated during EndMT identified in the meta-analysis.

Tumour type	Endothelial-related gene	Log2(Fold Change)	P values	tumour type	Endothelial-related gene	Log2(Fold Change)	P values	Tumour type	Endothelial-related gene	Log2(Fold Change)	P values
Breast invasive carcinoma	PECAM1	-1,615	1,00E-12	Lung adenocarcinoma	PECAM1	-2,391	1,62E-12	Bladder urothelial carcinoma	CD34	-2,520	6,01E-05
	VWF	-2,205	1,77E-05		VWF	-2,415	7,44E-15		CLDN5	-2,568	4,05E-04
	CD34	-2,167	1,62E-12		Tie1	-2,148	1,11E-16		MCAM	-1,933	4,52E-03
	CDH5	-1,756	1,00E-12		CD34	-1,627	1,63E-12		CLDN11	-2,652	8,74E-03
	CLDN5	-3,170	1,00E-12		CDH5	-2,856	1,00E-12				
	MCAM	-1,763	1,00E-12		CLDN5	-3,023	5,47E-14				
	CLDN11	-3,545	1,62E-12		CLDN11	-2,227	1,61E-09				
Colon adenocarcinoma	CLDN5	-2,891	1,67E-12	Uterine corpus endometrial carcinoma	PECAM1	-2,044	1,66E-05				
	CLDN11	-2,699	5,77E-05		KDR	-2,913	4,41E-07				
Renal clear cell carcinoma	OCLN	-2,799	8,18E-13		Tie1	-2,655	6,30E-08				
	CLDN11	-3,181	2,22E-10		CD34	-2,488	1,42E-07				
Rectum adenocarcinoma	CLDN5	-2,158	6,94E-07		CDH5	-1,828	1,81E-06				
					CLDN5	-2,651	3,10E-06				
					MCAM	-2,18	2,73E-06				

**Table 14:** Meta-analysis prognostic values

Tumour type	Endothelial-like marker	P value		
		Poor OS (Overall Survival)	Poor RFS (Relapse Free Survival)	FP (First Progression)
Colon adenocarcinoma	High expression of ANGPT2	3,30E-05	3,40E-04	NA
Breast invasive carcinoma	High expression of ANGPT2	2,20E-03	NS	NA
	Low expression of MCAM	1,50E-07	<1E-16	NA
	Low expression of CLDN11	1,90E-03	7,60E-07	NA
Lung adenocarcinoma	Low expression of PECAM1	4,20E-08	NA	3.7E-07
	Low expression of VWF	8,30E-03	NA	2,80E-03
	Low expression of CLDN5	1,40E-04	NA	1,70E-02
	Low expression of CDH5	3,00E-06	NA	1.3E-05
	Low expression of CLDN11	4,50E-03	NA	NS
Stomach adenocarcinoma	High expression of ANGPT2	3,50E-04	NA	1,90E-03

**Table 15:** Summary of the research gaps identified in this study

Research gaps identified in this study
Limited/lack of experimental studies focusing on certain solid cancer types (i.e. gastric cancer)
Limited information about the induction of EndMT in tumour-associated lymphatic endothelial cells
Reduced data about the capacity of chemoresistant cancerous cells to induce EndMT
Limited experimental information regarding the relationship between EMT in tumour cells and EndMT in endothelial cells
Lck of studies addressing the capacity of CSCs to induce the EndMT process
Reduced evidence about the ability of endothelial cells undergoing EndMT to promote a stem-like phenotype in cancerous cells
Reduced number of articles exploring the interaction between endothelial cells undergoing EndMT and stromal cells within the TME (i.e. CAFs, immune cells)
Few studies linking EndMT to microenvironmental conditions closely associated to cancer biology (i.e. altered blood flow, hypoxia)
Little information about the role of EndMT to promote the transition of endothelial cells towards non-fibroblastic mesenchymal-like cells
Few studies focusing on the reversibility of EndMT and on the relevance of MEndT process in cancer

## Abbreviations

BAEC: Bovine aortic endothelial cells
CAFs: Cancer-associated fibroblasts
CI: Confidence intervals
CLDN11: Claudin 11
CSCs: Cancer stem cells
EMT: Epithelial-to-mesenchymal transition
EndEMT: Endothelial-to-mesenchymal transition
EndMT: Endothelial-to-mesenchymal transition
End-myoT: Myofibroblast-like endothelial cell transition
End-N-myoT: Non-myofibroblast-like EndMT
FAPα: Fibroblast-associated protein alpha
FSP1: Fibroblast-specific protein 1
HBMEC: Human brain microvascular endothelial cells
HEMEC: Human endothelial microvascular cells
HIMEC: Human intestinal microvascular endothelial cells
HMEC1: Human microvascular endothelial cells
HMVEC-L: Human lung microvascular endothelial cells
HR: Hazard ratios
HUVECs: Human umbilical vein endothelial cells
IBMECs: Induced pluripotent stem cell-derived brain microvascular endothelial cells
MET: Mesenchymal-epithelial transition
MMP: Matrix Metalloproteinase
OHAT: Office of Health Assessment and Translation
PRISMA: Preferred Reporting Items for Systematic Reviews and Meta-Analyses
qPCR: Quantitative PCR
RBEC: Rat brain endothelial cells
SYRCLE: Systematic Review Center for Laboratory Animal Experimentation
TAMs: Tumour-associated macrophages
TECs: Tumour-associated endothelial cells
TME: Tumour microenvironment
αSMA: α-smooth muscle actin

## References

[1] Li Z (2024). Global, regional, and national burdens of early onset pancreatic cancer in adolescents and adults aged 15-49 years from 1990 to 2019 based on the Global Burden of Disease Study 2019: a cross-sectional study. Int J Surg.

[2] Kim H J, Kim Y H (2024). Molecular Frontiers in Melanoma: Pathogenesis, Diagnosis, and Therapeutic Advances. Int J Mol Sci.

[3] Wijayabahu A T (2024). Uterine cancer incidence trends and 5-year relative survival by race/ethnicity and histology among womwn under 50 years. Am J Obstet Gynecol.

[4] Toledo B (2022). Dual role of fibroblasts educated by tumour in cancer behavior and therapeutic perspectives. Int J Mol Cancer.

[5] Zhang Y (2024). An NFAT1-C3a-C3aR positive feedback loop in tumour-associated macrophages promotes a glioma stem cell malignant phenotype. Cancer Immunol Res.

[6] Zhang L (2024). Endothelial DGKG promotes tumour angiogenesis and immune evasion in hepatocellular carcinoma. J Hepatol.

[7] Han Y, Kang Y (2024). Phenotypic plasticity - implications for tumours in bone. J Bone Oncol.

[8] Zhang X, Wang W, Mo S, Sun X (2024). DEAD-box helicase 17 circRNA (circDDX17) reduces sorafenib resistance and tumourigenesis in hepatocellular carcinoma. Dig Dis Sci.

[9] Feigelman G, Simanovich E, Brockmeyer P, Rahat MA (2024). EMMPRIN promotes spheroid organization and metastatic formation: comparison between monolayers and spheroids of CT26 colon carcinoma cells. Front Immunol.

[10] Fan W (2024). DUSP5 regulated by YTHDF1-mediated m6A modification promotes epithelial-mesenchymal transition and EGFR-TKI resistance via the TGF-β/Smad signaling pathway in lung adenocarcinoma. Cancer Cell Int.

[11] Godina C (2024). High Caveolin-1 mRNA expression in triple-negative breast cancer is associated with an aggressive tumour microenvironment, chemoresistance, and poor clinical outcome. PLoS One.

[12] Zhang J (2024). ZNF263 cooperates with ZNF31 to promote drug resistance and EMT of pancreatic cancer through transactivating RNF126. J Cell Physio.

[13] Zhao Y (2024). circNOX4 activates an inflammatory fibroblast niche to promote tumour growth and metastasis in NSCLC via FAP/IL-6 axis. Mol Cancer.

[14] Xie DK (2024). Phenotypic comparison and the potential antitumour function of immortalized bone marrow-derived macrophages (iBMDMs). Front Immunol.

[15] Zhao Y (2023). Exosomal miR-218 derived from mesenchymal stem cells inhibits endothelial-to-mesenchymal transition by epigenetically modulating of BMP2 in pulmonary fibrosis. Cell Biol Toxicol.

[16] Li Y (2024). Transcriptomic signatures of individual cell types in cerebral cavernous malformation. Cell Commun Signal.

[17] Ying T (2023). Adropin inhibits the progression of atherosclerosis in ApoE-/-/Enho-/- mice by regulating endothelial-to-mesenchymal transition. Cell Death Discov.

[18] Xing Y (2024). Endothelial phosphodiesterase 4B inactivation ameliorates endothelial-to-mesenchymal transition and pulmonary hypertension. Acta Pharm Sin B.

[19] Clere N, Renault S, Corre I (2020). Endothelial-to-Mesenchymal Transition in Cancer. Front Cell Dev Biol.

[20] Ngo MT, Karvelis E, Harley BAC (2020). Multidimensional hydrogel models reveal endothelial network angiocrine signals increase glioblastoma cell number, invasion, and temozolomide resistance. Integr Biol.

[21] Janes PW (2024). An Anti-VEGF-B Antibody Reduces Abnormal Tumour Vasculature and Enhances the Effects of Chemotherapy. Cancers (Basel).

[22] Natesh NR, Mogha P, Chen A, Antonia SJ, Varghese S (2024). Differential roles of normal and lung cancer-associated fibroblasts in microvascular network formation. APL Bioeng.

[23] Zeisberg EM, Potenta L, Xie L, Zeisberg M, Kalluri R (2007). Discovery of endothelial to mesenchymal transition as a source for carcinoma-associated fibroblasts. Cancer Res.

[24] Huang M (2016). c-Met-mediated endothelial plasticity drives aberrant vascularization and chemoresistance in glioblastoma. J Clin Invest.

[25] Krizbai IA (2015). Endothelial-mesenchymal transition of brain endothelial cells: possible role during metastatic extravasation. PLoS One.

[26] Page MJ (2021). The PRISMA 2020 statement: An updated guideline for reporting systematic reviews. BMJ.

[27] National Institute of Health. OHAT Risk of Bias Rating Tool for Human and Animal Studies. Bethesda, MD, USA; 2015.

[28] Hooijmans MM (2014). SYRCLE's risk of bias tool for animal studies. BMC Med Res Methodol.

[29] McGuinness LA, Higgins JP (2021). Risk-of-bias VISualization (robvis): An R package and Shiny web app for visualizing risk-of-bias assessments. Res Synth Methods.

[30] Peng J (2021). Sema4C modulates the migration of primary tumour-associated lymphatic endothelial cells via an ERK-mediated pathway. Exp Ther Med.

[31] Lai C (2024). Single-cell analysis extracted CAFs-related genes to establish online app to predict clinical outcome and radiotherapy prognosis of prostate cancer. Clin Transl Oncol.

[32] Qiu ZW (2024). Breaking physical barrier of fibrotic breast cancer for photodynamic immunotherapy by remodeling tumour extracellular matrix and reprogramming cancer-associated fibroblasts. ACS Nano.

[33] Zhou P, Du X, Jia W, Feng K, Zhang Y (2024). Engineered extracellular vesicles for targeted reprogramming of cancer-associated fibroblasts to potentiate therapy of pancreatic cancer. Signal Transduct Target Ther.

[34] Huang H (2024). Fibroblast subtypes in pancreatic cancer and pancreatitis: from mechanisms to therapeutic strategies. Cell Oncol.

[35] Wilkus-Adamczyk K, Brodaczewska K, Majewska A, Kieda C (2023). Microenvironment commits breast tumour ECs to dedifferentiation by micro-RNA-200-b-3p regulation and extracellular matrix remodeling. Front Cell Dev Biol.

[36] Nie L (2014). Endothelial-mesenchymal transition in normal human esophageal endothelial cells cocultured with esophageal adenocarcinoma cells: Role of IL-1β and TGF-2. Am J Physiol Cell Physiol.

[37] Li DK (2022). Exosomal HMGA2 protein from EBV-positive NPC cells destroys vascular endothelial barriers and induces endothelial-to-mesenchymal transition to promote metastasis. Cancer Gene Ther.

[38] Morgos DT (2024). Targeting PI3K/AKT/mTOR and MAPK Signaling Pathways in Gastric Cancer. INT J MOL Sci.

[39] Hussain M (2024). Metastatic Hormone-Sensitive Prostate Cancer and Combination Treatment Outcomes: A Review. JAMA Oncol.

[40] Monda S (2024). Secondary Bladder Cancer After Prostate Cancer Treatment: An Age-matched Comparison Between Radiation and Surgery. Eur Urol Focus.

[41] Choi SH (2018). Tumour-vasculature development via endothelial-to-mesenchymal transition after radiotherapy controls CD44v6+ cancer cell and macrophage polarization. Nat Commun.

[42] Nagai N (2018). Downregulation of ERG and FLI1 expression in endothelial cells triggers endothelial-to-mesenchymal transition. PLoS Genet.

[43] Wei WF (2023). Cancer-associated fibroblast-derived PAI-1 promotes lymphatic metastasis via the induction of EndoMT in lymphatic endothelial cells. J Exp Clin Cancer Res.

[44] Wang SH (2017). Tumour cell-derived WNT5B modulates in vitro lymphangiogenesis via induction of partial endothelial-mesenchymal transition of lymphatic endothelial cells. Oncogene.

[45] Choi SH (2017). Dickkopf-1 induces angiogenesis via VEGF receptor 2 regulation independent of the Wnt signaling pathway. Oncotarget.

[46] Omori K (2018). Lipocalin-type prostaglandin D synthase-derived PGD2 attenuates malignant properties of tumour endothelial cells. J Pathol.

[47] Anderberg C (2013). Deficiency for endoglin in tumour vasculature weakens the endothelial barrier to metastatic dissemination. J Exp Med.

[48] Kimoto A (2023). Exosomes in ascites from patients with human pancreatic cancer enhance remote metastasis partially through endothelial-mesenchymal transition. Pancreatology.

[49] Matkar PN (2016). Novel regulatory role of neuropilin-1 in endothelial-to-mesenchymal transition and fibrosis in pancreatic ductal adenocarcinoma. Oncotarget.

[50] Kobayashi M (2022). Transforming growth factor-β-induced secretion of extracellular vesicles from oral cancer cells evokes endothelial barrier instability via endothelial-mesenchymal transition. Inflamm Regen.

[51] Jiao K (2020). 27-Hydroxycholesterol-induced EndMT acts via STAT3 signaling to promote breast cancer cell migration by altering the tumour microenvironment. Cancer Biol Med.

[52] Ciszewski WM (2017). The ILK-MMP9-MRTF axis is crucial for EndMT differentiation of endothelial cells in a tumour microenvironment. Biochim Biophys Acta - Mol Cell Res.

[53] Yoshimatsu Y (2020). TNF-α enhances TGF-β-induced endothelial-to-mesenchymal transition via TGF-β signal augmentation. Cancer Sci.

[54] Zhen J (2021). The 14-3-3η/GSK-3β/β-catenin complex regulates EndMT induced by 27-hydroxycholesterol in HUVECs and promotes the migration of breast cancer cells. Cell Biol Toxicol.

[55] Wang M (2024). Neovascularization directed by CAVIN1/CCBE1/VEGFC confers TMZ-resistance in glioblastoma. Cancer Lett.

[56] Gao F (2024). TSP50 facilitates breast cancer stem cell-like properties maintenance and epithelial-mesenchymal transition via PI3K p110α mediated activation of AKT signaling pathway. J Exp Clin Cancer Res.

[57] Kim SH, Song Y, Seo HR (2019). GSK-3β regulates the endothelial-to-mesenchymal transition via reciprocal crosstalk between NSCLC cells and HUVECs in multicellular tumour spheroid models. J Exp Clin Cancer Res.

[58] Fan CS (2018). Osteopontin-integrin engagement induces HIF-1α-TCF12-mediated endothelial-mesenchymal transition to exacerbate colorectal cancer. Oncotarget.

[59] Wawro ME (2018). Invasive Colon Cancer Cells Induce Transdifferentiation of Endothelium to Cancer-Associated Fibroblasts through Microtubules Enriched in Tubulin-β3. Int J Mol Sci.

[60] Fan CS (2019). Endothelial-mesenchymal transition harnesses HSP90α-secreting M2-macrophages to exacerbate pancreatic ductal adenocarcinoma. J Hematol Oncol.

[61] Ji HZ (2023). CXCL8 Promotes Endothelial-to-Mesenchymal Transition of Endothelial Cells and Protects Cells from Erastin-Induced Ferroptosis via CXCR2-Mediated Activation of the NF-κB Signaling Pathway. Pharmaceuticals.

[62] Shefler I (2022). Tumour-Derived Extracellular Vesicles Induce CCL18 Production by Mast Cells: A Possible Link to Angiogenesis. Cells.

[63] Chen J (2021). A reciprocal feedback of miR-548ac/YB-1/Snail induces EndMT of HUVECs during acidity microenvironment. Cancer Cell Int.

[64] Yeon JH (2018). Cancer-derived exosomes trigger endothelial to mesenchymal transition followed by the induction of cancer-associated fibroblasts. Acta Biomater.

[65] Mina SG (2016). Shear stress magnitude and transforming growth factor-βeta 1 regulate endothelial to mesenchymal transformation in a three-dimensional culture microfluidic device. RSC Adv.

[66] Mina SG, Huang P, Murray BT, Mahler GJ (2017). The role of shear stress and altered tissue properties on endothelial to mesenchymal transformation and tumour-endothelial cell interaction. Biomicrofluidics.

[67] Doerr M, Morrison J, Bergeron L, Coomber BL, Viloria-Petit A (2016). Differential effect of hypoxia on early endothelial-mesenchymal transition response to transforming growth beta isoforms 1 and 2. Microvasc Res.

[68] Smeda M (2022). Endothelial-mesenchymal transition induced by metastatic 4T1 breast cancer cells in pulmonary endothelium in aged mice. Front Mol Biosci.

[69] Wu Q (2023). PECAM-1 drives β-catenin-mediated EndMT via internalization in colon cancer with diabetes mellitus. Cell Commun Signal.

[70] Zhu K (2014). MiR-302c inhibits tumour growth of hepatocellular carcinoma by suppressing the endothelial-mesenchymal transition of endothelial cells. Sci Rep.

[71] Kim J (2020). Three-dimensional human liver-chip emulating premetastatic niche formation by breast cancer-derived extracellular vesicles. ACS Nano.

[72] Adjuto-Saccone M (2021). TNF-α induces endothelial-mesenchymal transition promoting stromal development of pancreatic adenocarcinoma. Cell Death Dis.

[73] Wu D (2021). TGF-β1-mediated exosomal lnc-MMP2-2 increases blood-brain barrier permeability via the miRNA-1207-5p/EPB41L5 axis to promote non-small cell lung cancer brain metastasis. Cell Death Dis.

[74] Motallebnejad P, Rajesh VV, Azarin SM (2022). Evaluating the Role of IL-1β in Transmigration of Triple Negative Breast Cancer Cells Across the Brain Endothelium. Cell Mol Bioeng.

[75] Wu DM (2020). PLEK2 mediates metastasis and vascular invasion via the ubiquitin-dependent degradation of SHIP2 in non-small cell lung cancer. Int J Cancer.

[76] Garcia J (2012). Tie1 deficiency induces endothelial-mesenchymal transition. EMBO Rep.

[77] Smeda M (2018). Nitric oxide deficiency and endothelial-mesenchymal transition of pulmonary endothelium in the progression of 4T1 metastatic breast cancer in mice. Breast Cancer Res.

[78] Choi SH (2016). HSPB1 inhibits the endothelial-to-mesenchymal transition to suppress pulmonary fibrosis and lung tumourigenesis. Cancer Res.

[79] Zhou H (2024). Integrating single-cell and spatial analysis reveals MUC1-mediated cellular crosstalk in mucinous colorectal adenocarcinoma. Clin Transl Med.

[80] Ghiabi P (2015). Breast cancer cells promote a notch-dependent mesenchymal phenotype in endothelial cells participating to a pro-tumoural niche. J Transl Med.

[81] Platel V (2022). NOX1 and NOX2: Two enzymes that promote endothelial-to-mesenchymal transition induced by melanoma conditioned media. Pharmacol Res.

[82] Yamada NO, Heishima K, Akao Y, Senda T (2019). Extracellular vesicles containing microRNA-92a-3p facilitate partial endothelial-mesenchymal transition and angiogenesis in endothelial cells. Int J Mol Sci.

[83] Takahashi K (2024). CD40 is expressed in the subsets of endothelial cells undergoing partial endothelial-mesenchymal transition in tumour microenvironment. Cancer Sci.

[84] Li ZX (2022). TGF-β1 promotes human breast cancer angiogenesis and malignant behavior by regulating endothelial-mesenchymal transition. Front Oncol.

[85] Tanaka M (2016). The endothelial adrenomedullin-RAMP2 system regulates vascular integrity and suppresses tumour metastasis. Cardiovasc Res.

[86] Magrini E (2014). Endothelial deficiency of L1 reduces tumour angiogenesis and promotes vessel normalization. J Clin Invest.

[87] Akatsu Y (2019). Fibroblast growth factor signals regulate transforming growth factor-β-induced endothelial-to-myofibroblast transition of tumour endothelial cells via Elk1. Mol Oncol.

[88] Verhulst E, Garnier D, De Meester I, Bauvois B (2022). Validating Cell Surface Proteases as Drug Targets for Cancer Therapy: What Do We Know, and Where Do We Go?. Cancers (Basel).

[89] Warden C, Wu X (2024). Critical Differential Expression Assessment for Individual Bulk RNA-Seq Projects. bioRxiv.

[90] Peart MJ (2005). Identification and functional significance of genes regulated by structurally different histone deacetylase inhibitors. Proceedings of the National Academy of Sciences, USA.

[91] Cardoso TF (2017). RNA-seq based detection of differentially expressed genes in the skeletal muscle of Duroc pigs with distinct lipid profiles. Scientific Reports.

[92] Zhao Q, Yu H, Shi M, Wang X, Fan Z, Wang Z (2024). Tumour microenvironment characteristics of lipid metabolism reprogramming related to ferroptosis and EndMT influencing prognosis in gastric cancer. Int Immunopharmacol.

[93] Lai C (2024). Single-cell analysis extracted CAFs-related genes to establish online app to predict clinical outcome and radiotherapy prognosis of prostate cancer. Clin Transl Oncol.

[94] Qiu ZW (2024). Breaking Physical Barrier of Fibrotic Breast Cancer for Photodynamic Immunotherapy by Remodeling Tumour Extracellular Matrix and Reprogramming Cancer-Associated Fibroblasts. ACS Nano.

[95] Boyle ST, Poltavets V, Kular J (2020). ROCK-mediated selective activation of PERK signalling causes fibroblast reprogramming and tumour progression through a CRELD2-dependent mech-anism. Nat Cell Biol.

[96] Huang H, Lu W, Zhang X, Pan J, Cao F, Wen L (2024). Fibroblast subtypes in pancreatic cancer and pancreatitis: from mechanisms to therapeutic strategies. Cell Oncol.

[97] Li Y (2023). An HGF-dependent positive feedback loop between bladder cancer cells and fibroblasts mediates lymphangiogenesis and lymphatic metastasis. Cancer Commun.

[98] Xiao J (2024). Leptin-mediated suppression of lipoprotein lipase cleavage enhances lipid uptake and facilitates lymph node metastasis in gastric cancer. Cancer Commun.

[99] Guo J, Song Z, Muming AJ, Zhang H, Awut E (2024). Cysteine protease inhibitor S promotes lymph node metastasis of esophageal cancer cells via VEGF-MAPK/ERK-MMP9/2 pathway. Naunyn Schmiedebergs Arch Pharmacol.

[100] Goess R (2024). Lymph node examination and survival in resected pancreatic ductal adenocarcinoma: retrospective study. BJS Open.

[101] Lindmark G (2024). qRT-PCR analysis of CEACAM5, KLK6, SLC35D3, MUC2 and POSTN in colon cancer lymph nodes—An improved method for assessment of tumor stage and prognosis. Int. J. Cancer.

[102] Wang H (2024). Linagliptin`s impact on lymphatic barrier and lymphangiogenesis in oral cancer with high glucose. Oral Dis.

[103] Gillot L (2021). The pre-metastatic niche in lymph nodes: formation and characteristis. Cell Mol Life Sci.

[104] Sun X (2022). Diffuse Large B-Cell Lymphoma Promotes Endothelial-to-Mesenchymal Transition via WNT10A/Beta-Catenin/Snail Signaling. Front Oncol.

[105] Hernández-Camarero P, López-Ruiz E, Marchal JA, Perán M (2021). Cancer: a mirrored room between tumour bulk and tumour microenvironment. J Exp Clin Cancer Res.

[106] Itoh G (2022). Cancer-associated fibroblasts educate normal fibroblasts to facilitate cancer cell spreading and T-cell suppression. Mol Oncol.

[107] Zhang X, Sun X, Guo C, Li J, Liang G (2024). Cancer-associated fibroblast-associated gene IGFBP2 promotes glioma progression through induction of M2 macrophage polarization. Am J Physiol Cell Physiol.

[108] Li C (2024). Spatial and Single-Cell Transcriptomics Reveal a Cancer-Associated Fibroblast Subset in HNSCC That Restricts Infiltration and Antitumour Activity of CD8+ T Cells. Cancer Res.

[109] Yao L (2023). Cancer-associated fibroblasts impair the cytotoxic function of NK cells in gastric cancer by inducing ferroptosis via iron regulation. Redox Biol.

[110] Luo H (2022). Pan-cancer single-cell analysis reveals the heterogeneity and plasticity of cancer-associated fibroblasts in the tumour microenvironment. Nat Commun.

[111] Ferreira FU (2019). Endothelial cells tissue-specific origins affects their responsiveness to TGF-β2 during endothelial-to-mesenchymal transition. Int J Mol Sci.

[112] Xiao L, McCann JV, Dudley AC (2015). Isolation and culture expansion of tumour-specific endothelial cells. J Vis Exp.

[113] Sabbineni H, Verma A, Somanath PR (2018). Isoform-specific effects of transforming growth factor β on endothelial-to-mesenchymal transition. J Cell Physiol.

[114] Xiao L (2015). Tumour endothelial cells with distinct patterns of TGFβ-driven endothelial-to-mesenchymal transition. Cancer Res.

[115] Pinto MT (2018). Endothelial cells from different anatomical origin have distinct responses during SNAIL/TGF-β2-mediated endothelial-mesenchymal transition. Am J Transl Res.

[116] Pinto MT, Covas DT, Kashima S, Rodrigues CO (2016). Endothelial Mesenchymal Transition: Comparative Analysis of Different Induction Methods. Biol Proced Online.

[117] Cao Y (2024). Integrin β8 prevents pericyte-myofibroblast transition and renal fibrosis through inhibiting the TGF-β1/TGFBR1/Smad3 pathway in diabetic kidney disease. Transl Res.

[118] Chen W (2021). PRRX1 deficiency induces mesenchymal-epithelial transition through PITX2/miR-200-dependent SLUG/CTNNB1 regulation in hepatocellular carcinoma. Cancer Sci.

[119] Cai W (2021). PERK-eIF2α-ERK1/2 axis drives mesenchymal-endothelial transition of cancer-associated fibroblasts in pancreatic cancer. Cancer Lett.

[120] Merk L (2024). Blocking TGF-β and Epithelial-to-Mesenchymal Transition (EMT)-mediated activation of vessel-associated mural cells in glioblastoma impacts tumour angiogenesis. Free Neuropathol.

[121] Abbona A (2022). Effect of Eribulin on Angiogenesis and the Expression of Endothelial Adhesion Molecules. Anticancer Res.

[122] Zhuo D (2024). Nudifloside, a Secoiridoid Glucoside Derived from Callicarpa nudiflora, Inhibits Endothelial-to-Mesenchymal Transition and Angiogenesis in Endothelial Cells by Suppressing Ezrin Phosphorylation. J Cancer.

[123] Choi KJ (2020). Endothelial-to-mesenchymal transition in anticancer therapy and normal tissue damage. Exp Mol Med.

[124] Shi X (2022). TGF-β signaling in the tumour metabolic microenvironment and targeted therapies. BioMed Central.

[125] Yang B (2017). Protein-altering and regulatory genetic variants near GATA4 implicated in bicuspid aortic valve. Nat Commun.

[126] Bhattarai P (2018). Recent advances in anti-angiogenic nanomedicines for cancer therapy. Nanoscale.

[127] Maruhashi R (2019). Chrysin enhances anticancer drug-induced toxicity mediated by the reduction of claudin-1 and 11 expression in a spheroid culture model of lung squamous cell carcinoma cells. Sci Rep.

[128] Kubelt C (2024). Temporal and regional expression changes and co-staining patterns of metabolic and stemness-related markers during glioblastoma progression. Eur J Neurosci.

[129] Bao X (2019). A novel epigenetic signature for overall survival prediction in patiens with breast cancer. J Transl Med.

[130] Najibi AJ (2022). Chemotherapy Dose Shapes the Expression of Immune-Interacting Markers on Cancer Cells. Cell Mol Bioeng.

