# Supplementary material for: What is the Impact of Endothelial-to-Mesenchymal Transition in Solid Tumours: A Qualitative Systematic Review and Quantitative Meta-Analysis
Source: Int J Biol Sci. 2025 Feb 24;21(5):2155–78. doi: 10.7150/ijbs.107045 (PMC11900810; doi:10.7150/ijbs.107045)
Supplement: Supplementary file 1 — Supplementary tables. [file ijbsv21p2155s1.pdf]

Supplementary Table S1: Meta-analysis of EndMT-related biomarkers expression changes.

Phenotype	Biomarker	Gene	Breast Invasive Carcinoma // Control (n=114) Y Tumor (n=1097)		Colon Adenocarcinoma // Control (n=41) Y Tumor (n=286)		Pancreatic Adenocarcinoma // Control (n=4) Y Tumor (n=178)		Lung Adenocarcinoma // Control (n=59) Y Tumor (n=515)		Prostate Adenocarcinoma // Control (n=52) Y Tumor (n=497)		Bladder Urothelial Carcinoma // Control (n=19) Y Tumor (n=408)		Esophageal Carcinoma // Control (n=11) Y Tumor (n=184)		Kidney renal clear cell carcinoma // Control (n=11) Y Tumor (n=184)		Glioblastoma multiforme // Control (n=5) Y Tumor (n=156)		Rectum adenocarcinoma // Control (n=10) Y Tumor (n=166)		Stomach adenocarcinoma // Control (n=34) Y Tumor (n=415)		Thyroid carcinoma // Control (n=34) Y Tumor (n=415)		Uterine corpus endometrial carcinoma // Control (n=35) Y Tumor (n=546)		Liver hepatocellular carcinoma // Control (n=34) Y Tumor (n=415)	
			Log2(Fold Change)	Statistical Significance (p values)	Log2(Fold Change)	Statistical Significance (p values)	Log2(Fold Change)	Statistical Significance (p values)	Log2(Fold Change)	Statistical Significance (p values)	Log2(Fold Change)	Statistical Significance (p values)	Log2(Fold Change)	Statistical Significance (p values)	Log2(Fold Change)	Statistical Significance (p values)	Log2(Fold Change)	Statistical Significance (p values)	Log2(Fold Change)	Statistical Significance (p values)	Log2(Fold Change)	Statistical Significance (p values)	Log2(Fold Change)	Statistical Significance (p values)	Log2(Fold Change)	Statistical Significance (p values)	Log2(Fold Change)	Statistical Significance (p values)	Log2(Fold Change)	Statistical Significance (p values)
Endothelial phenotype Mesenchymal-	CD31	PECAM1	-1,615	1,00E-12	-0,538	2,95E-02	-0,608	2,02E-03	-2,391	1,62E-12	-0,072	7,75E-01	-1,421	3,40E-03	0,274	7,33E-01	1,466	1,00E-12	1,245	4,38E-05	-1,152	2,93E-02	0,198	4,22E-05	0,237	1,65E-07	-2,044	1,66E-05	1,266	1,62E-12
	VEGFR2	KDR	-1,289	1,00E-12	0,010	1,58E-02	-0,123	9,30E-01	-1,399	1,56E-05	-0,924	3,93E-05	-0,969	6,66E-02	-0,161	7,83E-01	0,947	1,62E-12	1,239	5,95E-05	-0,115	3,90E-01	0,981	1,16E-13	-0,259	1,51E-01	-2,913	4,41E-07	-0,179	2,90E-01
	Tie2	AN	-0,5	1,62E-	1,633	1,00E-	0,123	4,45E-	0,516	2,04E-	-0,0	8,04E-	0,107	1,17E-	2,502	2,21E-	3,476	1,00E-	4,303	1,44E-	1,069	1,63E-	2,151	4,24E-	0,156	6,24E-	-0,3	3,88E-	1,946	1,11E-

like phenotype		GPT2	78	12		12		01		01	76	01		02		05		12		11		12		13		11	60	01		16
	VWF	VWF	-2,205	1,77E-05	-0,293	3,57E-01	0,085	7,99E-01	-2,415	7,44E-15	-0,043	5,26E-01	-1,050	7,33E-03	0,242	5,23E-01	3,588	1,62E-12	1,469	8,70E-08	-0,837	5,91E-01	0,728	1,44E-04	0,153	1,94E-07	-1,082	6,44E-04	2,399	1,00E-12
	Tie1	TIE1	-1,346	1,00E-12	-0,491	8,29E-01	-0,446	4,57E-01	-2,148	1,11E-16	-0,238	1,51E-01	-1,149	5,59E-04	0,374	7,24E-01	0,502	1,14E-09	0,431	4,97E-04	-0,595	6,39E-01	0,653	1,61E-07	-0,114	8,17E-01	-2,655	6,30E-08	0,503	1,64E-12
	CD34	CD34	-2,167	1,62E-12	-0,461	2,59E-02	-0,155	9,75E-01	-1,627	1,63E-12	-0,499	2,40E-03	-2,520	6,01E-05	0,635	3,31E-01	0,542	1,62E-12	0,858	2,25E-04	-1,010	6,78E-04	0,258	9,86E-01	-0,230	5,14E-01	-2,488	1,42E-07	2,755	1,62E-12
	ENOS	NOS3	-0,652	2,15E-06	1,089	4,44E-16	-0,344	4,38E-01	-0,394	4,92E-02	0,328	1,70E-03	-0,656	2,65E-02	0,257	7,95E-01	0,399	4,76E-03	0,045	4,53E-01	1,312	1,24E-06	1,283	1,21E-12	0,853	1,62E-12	-14,101	2,71E-02	-0,189	5,55E-04
	VE-Cadherin/CD144	CDH5	-1,756	1,00E-12	-0,294	7,85E-01	-0,123	8,45E-01	-2,856	1,00E-12	-0,007	1,28E-01	-1,109	1,01E-04	0,265	6,95E-01	1,209	1,62E-12	0,976	1,57E-01	-0,437	7,49E-01	0,866	4,49E-08	-0,123	2,16E-01	-1,828	1,81E-06	0,329	1,23E-06
	Zo1	TJP1	-0,748	1,00E-12	-0,290	1,86E-02	-0,114	6,98E-01	-1,019	2,20E-12	0,158	3,07E-02	-0,732	3,46E-06	0,449	1,77E-01	-0,610	8,18E-13	0,122	4,57E-01	-0,883	4,07E-02	0,444	8,86E-01	-0,136	4,58E-01	-1,082	5,54E-07	0,464	1,62E-12
	Claudin5	CLDN5	-3,170	1,00E-12	-2,891	1,67E-12	0,261	4,79E-01	-3,023	5,47E-14	-0,518	1,30E-02	-2,568	4,05E-04	-0,716	4,20E-04	0,038	2,15E-01	0,746	5,26E-09	-2,158	6,94E-07	-1,249	2,15E-06	-0,718	5,50E-03	-2,651	3,10E-06	0,446	3,61E-07
	MCAM	MCAM	-1,763	1,00E-12	0,136	2,33E-01	-0,487	4,15E-01	-0,909	2,04E-08	-1,033	3,58E-07	-1,933	4,52E-03	0,644	1,09E-01	2,292	1,62E-12	0,987	4,14E-01	-1,187	4,72E-02	0,013	4,30E-02	0,942	1,00E-12	-2,180	2,73E-06	1,728	1,62E-12
	Claudina11	CLDN11	-3,545	1,62E-12	-2,699	5,77E-05	-0,877	9,62E-01	-2,227	1,61E-09	-1,100	3,97E-07	-2,652	8,74E-03	-0,546	4,39E-01	-3,181	2,22E-10	-2,272	3,27E-01	-2,732	1,76E-02	-0,671	4,55E-02	0,272	3,02E-04	-2,150	4,66E-02	-0,540	5,13E-02

	Zo2	TJP2	-0,571	1,62E-12	0,194	3,06E-08	-0,040	3,31E-01	-0,948	1,63E-12	-0,336	2,96E-06	-0,474	9,40E-03	0,038	1,47E-01	-0,801	1,00E-12	-0,685	3,16E-01	0,491	3,34E-02	0,131	1,37E-02	-0,078	7,57E-01	0,308	2,43E-01	-0,316	7,19E-02
	Occludin1	OCLN	0,513	1,62E-12	-0,460	1,60E-02	-0,439	7,49E-01	-0,384	8,46E-01	0,285	2,25E-09	0,267	8,79E-01	0,380	6,91E-01	-2,799	1,62E-12	-0,161	8,53E-01	0,102	5,49E-01	1,011	7,50E-12	-0,496	4,59E-04	2,063	1,57E-05	0,493	9,44E-15
	αSMA	ACTA2	-2,042	1,62E-12	-0,778	4,10E-02	-0,316	8,14E-01	-0,322	8,89E-02	-1,422	2,65E-08	-3,923	1,36E-03	-0,587	3,03E-02	1,241	5,90E-05	2,322	1,29E-07	-2,454	4,28E-03	-0,894	7,04E-03	0,495	6,19E-08	-4,471	1,01E-06	1,119	1,00E-12
	FSP1	AIFM2	-1,326	1,63E-12	-0,104	4,18E-01	0,012	5,29E-01	0,559	1,00E-12	-0,925	3,89E-12	-0,015	7,91E-02	0,264	1,65E-01	0,128	7,56E-03	-0,047	4,24E-01	0,223	2,37E-01	0,511	6,44E-15	-0,530	1,37E-12	0,702	1,00E-12	1,328	1,00E-12
	Vimentin	VIM	-1,369	1,62E-12	-0,825	3,62E-04	-0,762	1,69E-01	-1,254	1,00E-12	-0,540	6,48E-05	-1,959	1,92E-03	1,136	2,48E-03	2,878	1,62E-12	3,463	1,62E-12	-1,185	9,60E-04	-0,213	7,80E-01	-0,198	5,22E-02	-1,347	1,67E-05	0,683	1,16E-11
	N-Cadherin	CDH2	1,610	3,45E-08	-0,405	9,01E-01	-1,502	7,97E-01	0,419	1,27E-08	-2,091	2,50E-02	-0,056	2,33E-01	-4,383	2,82E-03	1,329	1,00E-12	0,601	1,34E-08	-2,650	5,34E-02	-0,070	7,92E-01	3,142	1,00E-12	-1,771	6,78E-01	0,027	4,17E-05
	SM22α	TAGLN	-1,591	1,00E-12	-1,732	1,98E-02	-0,335	7,05E-01	-0,815	1,44E-03	-1,511	3,82E-07	-3,555	3,46E-03	-0,754	6,02E-03	0,133	9,71E-01	1,582	1,62E-12	-4,243	3,44E-03	-1,307	3,82E-03	-0,456	8,16E-02	-4,305	3,13E-06	0,227	1,62E-02
	FAPα	FAP	1,759	1,62E-12	4,484	1,00E-12	-0,787	9,89E-01	2,165	1,62E-12	1,281	4,05E-10	0,794	1,64E-12	4,786	2,84E-07	2,716	3,42E-04	-8,753	4,82E-07	1,879	2,62E-11	4,202	1,62E-12	0,391	1,62E-12	-2,430	9,57E-05	3,735	1,62E-12
	CD44	CD44	-0,249	9,79E-05	1,395	1,62E-12	-0,709	2,82E-01	-1,100	1,00E-12	-0,354	1,44E-03	-0,742	6,08E-01	2,544	2,17E-01	1,193	3,27E-13	2,823	6,33E-07	0,974	1,47E-07	0,640	9,92E-07	1,062	1,62E-12	-1,015	6,24E-03	0,175	9,19E-09
	SNAIL	SNAI1	-0,066	9,72E-01	1,701	1,62E-12	-0,375	6,73E-01	-0,451	3,01E-02	-0,411	4,39E-02	-1,040	1,04E-01	2,356	2,68E-09	0,747	2,96E-07	0,056	5,84E-01	1,821	6,08E-08	1,315	1,51E-11	-2,016	1,62E-12	-1,247	4,05E-04	-0,717	8,52E-01
	Slug	SN	-	1,7	-	1,9	-	6,3	-	4,0	-	1,1	-	8,1	1,9	6,3	-	1,3	1,7	9,7	-	1,0	0,4	6,1	-	1,7	-	6,6	0,9	3,6

		AI2	1,093	4E-12	0,584	6E-01	0,022	5E-01	0,354	6E-03	2,096	7E-08	0,338	2E-02	87	3E-01	0,223	5E-01	06	1E-08	0,746	2E-01	10	1E-05	0,584	8E-02	2,913	2E-07	36	7E-13
	Zeb1	ZEB1	-1,127	1,63E-12	-0,702	3,20E-03	0,107	2,18E-01	-1,656	1,63E-12	-0,946	3,84E-06	-2,987	1,92E-03	0,592	2,39E-07	0,467	2,03E-14	0,845	6,44E-02	-3,017	1,41E-02	0,364	2,77E-02	-0,881	1,65E-12	-3,928	1,65E-09	0,317	5,02E-12
	TWIST1	TWIST1	-1,557	7,72E-12	1,543	1,11E-16	-0,578	7,02E-01	1,649	1,62E-12	1,588	1,00E-12	-2,058	2,84E-02	1,287	1,76E-02	0,802	5,90E-05	0,761	6,19E-04	0,227	7,55E-08	0,281	5,63E-01	-0,209	2,72E-02	-2,055	8,24E-03	0,365	8,82E-03
	Fibronectin	FN1	2,890	1,00E-12	1,053	1,40E-04	-0,604	7,14E-01	-0,634	5,18E-01	-0,223	1,60E-02	-0,815	8,23E-01	1,043	9,78E-01	1,698	1,00E-12	3,217	1,00E-12	-0,668	5,15E-01	1,749	4,08E-03	5,761	1,00E-12	-1,039	3,32E-02	0,221	2,14E-07
	Collagen	COL1A1	2,583	1,62E-12	2,383	1,00E-12	-0,708	7,05E-01	3,018	1,00E-12	0,443	2,52E-03	-0,271	3,61E-01	2,910	9,10E-11	3,139	1,09E-13	4,011	4,69E-06	1,452	3,20E-13	2,782	1,62E-12	1,268	3,33E-11	-0,221	4,93E-02	1,156	1,24E-14
	Collagen	COL1A2	1,438	1,62E-12	1,489	1,00E-12	-0,529	6,88E-01	1,226	1,00E-12	-0,081	3,13E-01	-1,085	8,30E-01	2,233	3,52E-02	1,732	3,33E-16	3,134	1,20E-04	0,522	1,73E-05	2,248	1,62E-12	0,256	7,99E-08	-1,322	1,24E-03	1,252	1,64E-12
	Matrix metalloproteinase	MMP2	-0,278	2,31E-01	0,002	2,14E-03	0,284	7,18E-01	0,270	3,54E-06	-0,697	2,16E-04	-1,701	1,50E-03	1,290	1,79E-01	0,192	1,01E-01	3,069	1,62E-12	-0,148	4,66E-02	0,288	1,84E-06	-0,280	1,54E-01	-1,718	6,84E-03	0,288	3,42E-04
	Matrix metalloproteinase	MMP9	2,993	2,09E-05	1,197	1,38E-10	-0,948	8,04E-01	2,361	1,62E-12	2,937	1,13E-07	1,915	7,45E-02	4,244	1,77E-01	3,267	1,34E-10	6,140	4,42E-12	1,047	9,03E-04	3,029	5,55E-16	0,315	1,86E-01	3,387	1,01E-09	1,567	2,73E-06
	TGFβ1	TGF	0,773	1,62E-	-0,0	6,94E-	-0,4	3,21E-	-0,4	1,28E-	-0,2	1,04E-	0,008	1,61E-	2,340	2,40E-	1,637	1,00E-	1,225	3,66E-	-0,2	3,36E-	0,162	2,92E-	1,820	1,62E-	-1,0	2,10E-	0,246	2,19E-

		B1		12	93	02	74	01	47	06	52	01		01		01		12		05	62	01		03		12	72	05		11
	Caldesmon	CALD1	-0,925	1,00E-12	-0,147	3,88E-02	-0,420	8,67E-01	-0,671	2,35E-04	-1,099	3,06E-08	-2,157	6,46E-03	0,572	2,48E-01	0,708	1,81E-11	2,187	1,00E-12	-2,762	1,19E-02	-0,186	1,76E-02	-0,163	8,56E-03	-3,032	5,85E-09	0,089	1,04E-04
	Calponin	CNN1	-4,032	1,62E-12	-3,735	2,14E-02	-0,359	6,61E-02	-1,814	2,67E-11	-1,441	1,18E-07	-6,255	3,10E-03	-3,232	8,28E-01	0,210	5,95E-01	-0,913	7,26E-01	-6,083	2,08E-03	-3,854	2,34E-03	-1,080	3,44E-03	-6,973	9,95E-07	1,018	1,86E-09
	Tropomyosin	TPM1	-0,698	4,84E-04	-0,759	2,76E-03	-1,333	1,05E-01	0,006	7,19E-07	-1,311	2,67E-07	-2,399	3,86E-03	0,607	2,32E-01	0,689	1,62E-12	-0,483	9,81E-01	-2,429	3,34E-03	-0,504	2,02E-03	-0,146	1,58E-01	-2,344	1,56E-06	1,144	5,02E-12

APPENDIX A. Selected article list

Reference	Publication year	Journal	DOI
Jiao et al. [44]	2020	Cancer Biology and Medicine	10.20892/j.issn.2095-3941.2019.0262
Chen et al. [56]	2021	Cancer Cell International	10.1186/s12935-021-02388-8
Ghiabi et al. [73]	2015	Journal of Translational Medicine	10.1186/s12967-015-0386-3
Huang et al. [21]	2016	Journal of Clinical Investigation	10.1172/JCI84876
Wei et al. [36]	2023	Journal of Experimental and Clinical Cancer Research	10.1186/s13046-023-02714-0
Yeon et al. [57]	2018	Acta Biomaterialia	10.1016/j.actbio.2018.07.001
Lin et al. [28]	2015	Oncotarget	10.18632/oncotarget.5325
Takahashi et al. [76]	2024	Cancer Science	10.1111/cas.16045
Ji et al. [54]	2023	Pharmaceuticals	10.3390/ph16091210
Anderberg et al. [40]	2013	Journal of Experimental Medicine	10.1084/jem.20120662
Doerr et al. [60]	2016	Microvascular Research	10.1016/j.mvr.2016.08.001
Choi et al. [38]	2017	Oncotarget	10.18632/oncotarget.19769
Zeisberg et al. [20]	2007	Cancer Research	10.1158/0008-5472.CAN-07-3127
Nagai et al. [35]	2018	PLoS Genetics	10.1371/journal.pgen.1007826
Magrini et al. [79]	2014	J Clin Invest	10.1172/JCI70683
Fan et al. [53]	2019	Journal of Hematology and Oncology	10.1186/s13045-019-0826-2
Nie et al. [33]	2014	American Journal of Physiology -	10.1152/ajpcell.000

		Cell Physiology	81.2014
Smeda et al. [61]	2022	Frontiers in Molecular Biosciences	10.3389/fmolb.2022.1050112
Krizbai et al. [22]	2015	PloS one	10.1371/journal.pone.0119655
Motallebnejad et al. [67]	2021	Cellular and molecular bioengineering	10.1007/s12195-021-00710-y
Li et al. [34]	2022	Cancer gene therapy	10.1038/s41417-022-00453-6
Kimoto et al. [41]	2023	Pancreatology	10.1016/j.pan.2023.04.002
Yamada et al. [75]	2019	International journal of molecular sciences	10.3390/ijms20184406.
Akatsu et al. [80]	2019	Molecular oncology	10.1002/1878-0261.12504
Kim et al. [50]	2019	Journal of experimental & clinical cancer research	10.1186/s13046-019-1050-1
Choi et al. [71]	2016	Cancer research	10.1158/0008-5472.CAN-15-0952
Zhou et al. [72]	2024	Clinical and translational medicine	10.1002/ctm2.1701
WaWro et al. [52]	2018	International journal of molecular sciences	10.3390/ijms20010053
Omori et al. [39]	2018	Journal of pathology	10.1002/path.4993
Adamczyk et al. [32]	2023	Frontiers in cell and developmental biology	10.3389/fcell.2023.1125077
Zhu et al. [63]	2014	Scientific reports	10.1038/srep05524
Wang et al. [48]	2024	Cancer letters	10.1016/j.canlet.2023.216593
Vonach et al. [29]	2011	British journal of cancer	10.1038/bjc.2011.194
Smeda et al. [70]	2018	Breast cancer research	10.1186/s13058-018-1013-z

Matkar et al. [42]	2016	Oncotarget	10.18632/oncotarget.11060
Platel et al. [74]	2022	Pharmacological research	10.1016/j.phrs.2022.106097
Fan et al. [51]	2017	Oncotarget	10.18632/oncotarget.23578
Wu et al. [62]	2023	Cell comunication and signaling	10.1186/s12964-023-01193-2
Wu et al. [68]	2020	International journal of cancer	10.1002/ijc.32675
Peng et al. [27]	2021	Experimental and therapeutic medicine	10.3892/etm.2021.10535
Mina et al. [58]	2016	RSC advances	10.1039/c6ra16607e
Wu et al. [66]	2021	Cell death & disease	10.1038/s41419-021-04004-z
Li et al. [77]	2022	Frontiers in oncology	10.3389/fonc.2022.1051148
Zhen et al. [47]	2021	Cell biology and toxicology	10.1007/s10565-020-09564-y
Tanaka et al. [78]	2016	Cardiovascular research	10.1093/cvr/cvw166
Ciszewski et al. [45]	2017	Biochimica et biophysica acta. Molecular cell research	10.1016/j.bbamcr.2017.09.004
Mina et al. [59]	2017	Biomicrofluidics	10.1063/1.4991738
Kim et al. [64]	2020	ACS nano	10.1021/acsnano.0c04778
Garcia et al. [69]	2012	EMBO reports	10.1038/embor.2012.29
Yoshimatsu et al. [46]	2020	Cancer science	10.1111/cas.14455
Adjuto-Saccone et al. [65]	2021	Cell death & disease	10.1038/s41419-021-03920-4
Kobayashi et al. [43]	2022	Inflammation and regeneration	10.1186/s41232-022-00225-7
Wang et al. [37]	2017	Oncogene	10.1038/onc.2016.3

			17
Shefler et al. [55]	2022	Cells	10.3390/cells11030353
